# Bioactivity Assessment and Untargeted Metabolomics of the Mediterranean Sea Pen *Pennatula phosphorea*

**DOI:** 10.3390/md23050218

**Published:** 2025-05-21

**Authors:** Silvia Scarpato, Daniel Venturi, Fortunato Palma Esposito, Maria Cristina Mangano, Gianluca Sarà, Francesco Margiotta, Ester Pagano, Maria Miraglia, Enrico Sangiovanni, Mercedes Garcia-Gil, Lorenzo Di Cesare Mannelli, Carla Ghelardini, Mario Dell’Agli, Angelo A. Izzo, Paola Nieri, Donatella de Pascale, Gerardo Della Sala

**Affiliations:** 1 Department of Ecosustainable Marine Biotechnology, Stazione Zoologica Anton Dohrn, Via A.F. Acton, Molosiglio, 80133 Naples, Italy; silvia.scarpato@szn.it (S.S.); donatella.depascale@szn.it (D.d.P.); 2 Department of Neuroscience, Psychology, Drug Research and Child Health-Neurofarba–Section of Pharmacology and Toxicology, University of Florence, 50139 Florence, Italy; daniel.venturi1@unifi.it (D.V.); francesco.margiotta@unifi.it (F.M.); lorenzo.mannelli@unifi.it (L.D.C.M.); carla.ghelardini@unifi.it (C.G.); 3 Department of Integrated Marine Ecology, Stazione Zoologica Anton Dohrn, Sicily Marine Centre, Lungomare Cristoforo Colombo (Complesso Roosevelt), 90142 Palermo, Italy; mariacristina.mangano@szn.it; 4 NBFC, National Biodiversity Future Center, Piazza Marina 61, 90133 Palermo, Italy; gianluca.sara@unipa.it; 5 Laboratory of Ecology, Department of Earth and Marine Sciences, DiSTeM, University of Palermo, Viale delle Scienze Ed. 16, 90128 Palermo, Italy; 6 Department of Pharmacy, School of Medicine and Surgery, University of Naples Federico II, 80138 Naples, Italy; ester.pagano@unina.it (E.P.); maria.miraglia2@unina.it (M.M.); aaizzo@unina.it (A.A.I.); 7 Department of Pharmacological and Biomolecular Sciences, University of Milan, 20133 Milan, Italy; enrico.sangiovanni@unimi.it (E.S.); mario.dellagli@unimi.it (M.D.); 8 Department of Biology, University of Pisa, 56127 Pisa, Italy; mercedes.garcia@unipi.it; 9 Department of Pharmacy, University of Pisa, 56126 Pisa, Italy; paola.nieri@unipi.it; 10 Interdepartmental Center of Marine Pharmacology (MarinePHARMA), University of Pisa, 56126 Pisa, Italy

**Keywords:** octocorals, *Pennatula phosphorea*, cisplatinum-induced cytotoxicity protection, anti-melanoma activity, anti-inflammatory effect, mass spectrometry, molecular networking, glycerophospholipids, phosphosphingolipids, prostaglandins

## Abstract

Octocorals have proven to be a prolific source of bioactive natural products, exhibiting a wide spectrum of pharmacological activities. Among octocorals, Pennatulaceans, commonly known as sea pens, are among the most dominant soft coral species living in benthic communities. Nonetheless, reports on bioactivity and chemical investigations of this genus are scarce. This prompted us to shed light on the pharmacological potential of the extracts of the sea pen *Pennatula phosphorea*, Linneus 1758, and gain an overview of its metabolome. Crude octocoral extracts, obtained with a modified Kupchan extraction protocol, were assessed for their bioactivity potential, revealing the hexanic extract to exert anti-inflammatory effects and interesting protective properties in an in vitro model of sarcopenia and in auditory HEI-OC1 cisplatin-treated cells, while the chloroformic extract was active in reducing A375 melanoma cell viability in a concentration-dependent manner. An untargeted metabolomic analysis unveiled that *P. phosphorea* collects a wide array of glycerophospholipids and phosphosphingolipids belonging to the ceramide phosphoinositol class, which were exclusive or more abundant in the hexanic extract. Their proven anti-inflammatory and cytoprotective effects could demonstrate the activity shown by the *P. phosphorea* hexanic extract. In addition, a group of prostaglandins, eluted mainly in the chloroformic extract, were putatively annotated. Since prostanoids from marine origin have been demonstrated to exert cytotoxic and anti-proliferative properties against various cancer cell lines, the presence of PGs in the *P. phosphorea* chloroform extract could justify its anti-melanoma activity. This is the first report on the presence of glycerophospholipids, phosphosphingolipids, and prostaglandins, along with the identification of novel congeners, in sea pens.

## 1. Introduction

Octocorals are major components of the benthic fauna of many coral reefs, occurring throughout the world’s oceans. Also known as octocorallia and belonging to the class Anthozoa, phylum Cnidaria, they are sessile marine polypoid animals. The name “octocoral” derives from their structural peculiarity of always bearing eight pinnulate tentacles [1]. With a sole known exception (*Taiaroa tauhou*), the majority of the species are colonial, meaning that each polyp is linked to a central gastro-vascular cavity, called coelenteron [2].

Unlike scleractinian or hard corals, the main reef-building corals, which usually deposit a massive exoskeleton of calcium carbonate, octocorals lack this physical protection. Relying heavily on chemical defence strategies to overcome competitive situations and to avoid overgrowth and fouling, they are known to store a variety of secondary metabolites in their bodies and/or release them into the surrounding environment [3]. Hence, soft corals are a rich source of bioactive natural products, including polyketides, alkaloids, sesqui- and diterpenoids, showing anti-microbial, anti-tumoral, anti-inflammatory, anti-viral, anti-malarial, and neuroprotective properties [4].

It is worth mentioning that a group of cembrene-type diterpenes, including flexibilide and sinulariolide, isolated from the soft coral *Sinularia flexibilis*, showed Gram-positive anti-microbial properties and anti-tumoral activities against HepA and EAC cells [5,6]. Noteworthily, a plethora of anti-cancer agents have been identified from the extracts of soft coral *Clavularia viridis*, including a series of prostanoids called clavulones and the steroids stoloniferone E and yonarasterols, displaying potent cytotoxicity against mouse lymphocytic leukemia (P-388) and human colorectal adenocarcinoma (DLD-1), respectively [6,7]. 

Octocorallia includes more than 3000 species and is further divided into three orders: Alcyonacea, Helioporacea, and Pennatulacea [8]. Among them, in the last years, the order Pennatulacea has demonstrated to be a promising reservoir of compounds for therapeutic uses [9].

Listing around 200 species, Pennatulaceae are commonly known as sea pens due to the resemblance of their body shape to a feather. Overall, each colony has a central stem consisting of a lower segment (peduncle) for anchoring the colony in the soft ocean floor and an upper part, the rachis, including specialized polyps, which develop into an erect stalk extended in the water column [10]. Sea pens play a crucial ecological role in deep-sea ecosystems, increasing biodiversity and providing important habitat for numerous fishes and invertebrates, which use them as a substrate and refuge for their eggs [11]. The majority of pennatulaceans live in the deep sea, but many species can also be found in shallow waters of the Indo-Pacific and Mediterranean basins [10,12].

Few studies report the isolation of new bioactive sea-pen-derived natural products, including three new briarane diterpenes, bathyptilone A-C, found in an Antarctic specimen of *Anthoptilum grandiflorum*, one of them showing selective cytotoxic activity against the neurogenic mammalian cell line Ntera-2 [13], the eunicellin-type diterpenoid, vigulariol, and a new sesquiterpenoid, junceol A, isolated from the sea pen *Vigularia juncea*, exhibiting cytotoxicity against human lung adenocarcinoma cells (A549) and mouse lymphocytic leukemia P-388 cancer cells, respectively [14,15]. Another example is the 2-acetoxy-verecynarmin C, a briarane diterpenoid isolated from a specimen of the sea pen *Pennatula aculeata*, showing COX-1/COX-2 inhibitory activity [16].

In this context, the exploration of the chemistry of pennatulaceans may lead to the discovery of a chemical armamentarium that is yet to be uncovered. This prompted us to shed light on the bioactivity potential of the extracts of the scarcely studied sea pen *Pennatula phosphorea*, Linneus 1758, and to investigate their metabolome. A panel of pharmacological assays was conducted to study the therapeutical potential of the *P. phosphorea* extracts showing, for the hexanic and chloroformic extracts, promising biological activities. Extracts of *P. phosphorea* were then analyzed with an untargeted metabolomic strategy combining high-resolution liquid chromatography coupled with tandem mass spectrometry (LC-HRMS^2^) and the feature-based molecular networking workflow, whose capacity to facilitate dereplication (i.e., early identification of already known natural products), as well as the discovery of unknown NPs, has been widely demonstrated [17,18,19]. 

## 2. Results

### 2.1. Collection and Extraction of the Octocoral P. phosphorea

Specimens of the octocoral *P. phosphorea* (Anthozoa: Octocorallia)*,* phosphorescent sea pen, collected along the continental shelf off Portopalo di Capo Passero (Southern Sicily Island, Central Mediterranean Sea) along fishing grounds and resulting from trawling by-catch fraction, were subjected to exhaustive extraction using a modified Kupchan extraction scheme. The afforded crude hexanic (70.7 mg), chloroformic (82.8 mg), and methanolic (624.3 mg) extracts were screened by six biological assays, described below.

### 2.2. Protective Effects on Muscular Cells

#### 2.2.1. Hexanic Extract of *P. phosphorea* Reduced Growth Inhibition in Dexamethasone-Treated C2C12 Myoblast Cells

**Figure 1:**
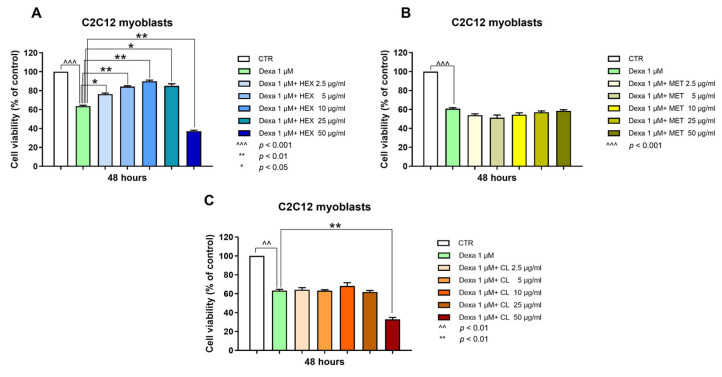
Cell viability analysis, MTT assay. Cell viability was assessed in the C2C12 cell line following a 48-hour treatment with three fractionated *P. phosphorea* extracts [hexane, HEX (**A**), methanol, MET (**B**) chloroform, CL (**C**)] at five distinct concentrations (2.5–50 μg/mL) in combination with Dexa (1 μM) using the MTT assay. All results are presented as a percentage relative to the control group, which was arbitrarily set at 100%. The data are reported as the mean ± S.E.M. based on six replicates for each experiment, and two independent experiments (n = 2) were conducted. Statistical analysis was carried out using non-parametric *t*-test, followed by a Bonferroni post hoc comparison using the GraphPad Prism 10 software. ^^^ *p* < 0.001; ^^ *p* < 0.01; ** *p* < 0.01; * *p* < 0.05.

C2C12 myoblast cells were treated with dexamethasone (Dexa) to reproduce an in vitro model of sarcopenia [20]. A reduction in viability of about 40% was reached using 1 μM Dexa. Higher concentrations did not increase the cell mortality rate. Therefore, we chose 1 μM Dexa for 48 h as standard damage. The hexanic extract of *P. phosphorea* (HEX) prevented Dexa-induced cell damage, increasing viability from 60% (Dexa) to about 90% (10 μg/mL HEX). HEX showed a significant effect starting from 2.5 μg/mL to 25 μg/mL. The 50 μg/mL concentration was toxic per se (Figure 1A). Chloroform (CL) and methanol (MET) extracts did not significantly modify the viability of Dexa-treated C2C12 myoblasts (Figure 1B,C). The highest concentration (50 μg/mL) of CL was toxic per se (Figure 1C).

#### 2.2.2. Hexanic Extract of *P. phosphorea* Reduced Cell Proliferation in Dexamethasone-Treated C2C12 Myoblast Cells

**Figure 2:**
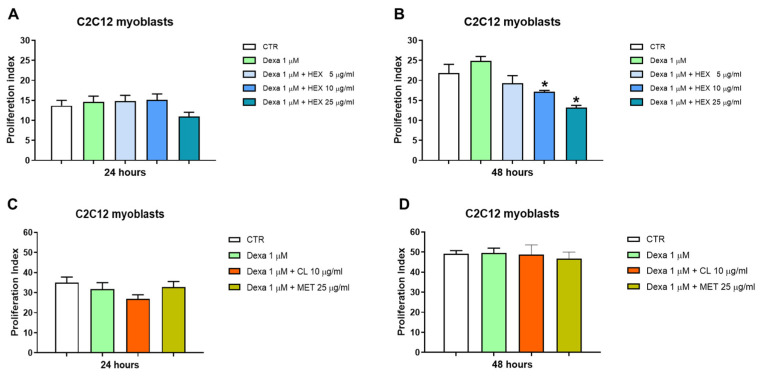
Cell proliferation analysis, CFSE assay. Cell proliferation was assessed in the C2C12 cell line following a 24 and 48 h treatment with three *P. phosphorea* extracts at different concentrations in combination with Dexa (1 μM): hexane, HEX 5, 10, 25 μg/mL (**A**,**B**), chloroform, CL 10 μg/mL and methanol, MET 25 μg/mL (**C**,**D**). The data are reported as the mean ± S.E.M. based on three replicates for each experiment, and two independent experiments (n = 2) were conducted. Statistical analysis was carried out using non-parametric *t*-test, followed by a Bonferroni post hoc comparison using the GraphPad Prism 10 software. * *p* < 0.05.

A significant reduction in cell proliferation was observed after treatment with higher concentrations of HEX (10 and 25 μg/mL) in combination with Dexa 1μM after 48 h of treatment (Figure 2B). This, in addition to a morphological change seen under the microscope, led us to hypothesize a possible anti-proliferative effect of the hexanic extract in favor of differentiation from myoblasts to myotubes (see Section 2.2.3). Dexa alone and in combination with MET and CL did not lead to a significant variation in cell proliferation in any of the two times analyzed (Figure 2C,D).

#### 2.2.3. Hexanic Extract of *P. phosphorea* Promoted the Differentiation of C2C12 Cells from Myoblasts to Myotubes

**Figure 3:**
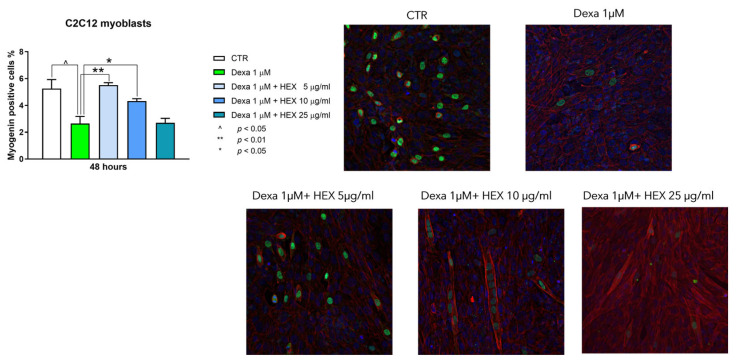
Immunofluorescence analysis on C2C12 cells treated for 48 h with HEX at three different concentrations (5, 10, 25 μg/mL) in combination with Dexa (1 μM). (**Left**) Representative chart of the percentage of myogenin-positive cells. (**Right**) Representative images of immunofluorescence: blue signal from nuclei obtained by DAPI staining, phalloidin staining red, and myogenin staining green. The data are reported as a percentage calculated by the ratio between the number of myogenin-positive cells and the total cells identified by DAPI (100%), based on at least four images for each experiment. Three independent experiments (n = 3) were conducted. Statistical analysis was carried out using non-parametric *t*-test, followed by a Bonferroni post hoc comparison using the GraphPad Prism 10 software. ^ *p* < 0.05; ** *p* < 0.01; * *p* < 0.05.

Myogenin is an early marker of differentiation that plays a pivotal role in the regulation of the fusion of myoblasts to form myotubes [21]. To analyze the impact of HEX on cell differentiation, the number of myogenin-positive cells was measured. Dexa (1 μM, 48 h, added during the differentiation period between 24 and 72 h) reduced the number of myogenin-positive cells by about 40%. Co-treatment with HEX has led to a complete restoration of myogenin expression at the lower concentration tested (5 μg/mL). Therefore, also considering the reduction in proliferation, the ability of HEX to promote the differentiation from myoblasts to myotubes is suggested (Figure 3).

#### 2.2.4. Hexanic Extract of *P. phosphorea* Balanced Apoptosis in Starved C2C12 Myoblast Cells

**Figure 4:**
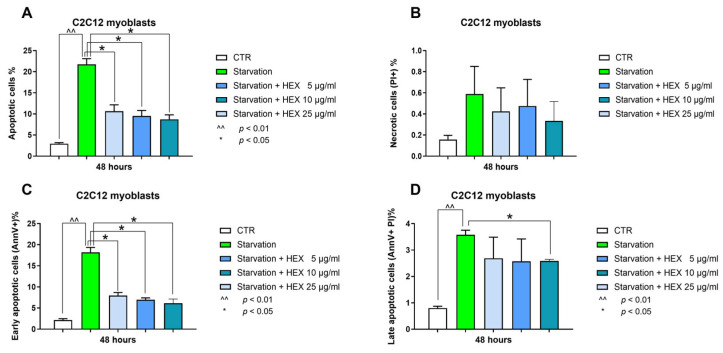
Annexin-V and PI assay. Apoptosis (total, early, and late) (panels **A**,**C**,**D**) and necrosis (panel **B**) analysis were assessed in the C2C12 cells treated with HEX at three different concentrations (5, 10, 25 μg/mL) in starvation medium. The data are reported as the mean ± S.E.M. based on three replicates for each experiment, and two independent experiments (n = 2) were conducted. Statistical analysis was carried out using non-parametric *t*-test, followed by a Bonferroni post hoc comparison using the GraphPad Prism 10 software. ^^ *p* < 0.01; * *p* < 0.05.

The anti-apoptotic properties of HEX were assessed by annexin V-fluorescein isothiocyanate and propidium iodide (PI) double staining. Starvation was used as pro-apoptotic damage. The 20%-increased number of apoptotic cells was prevented by the extract (Figure 4A). In particular, this protection occurred during the early stages of apoptosis (early apoptosis) with all concentrations tested (5, 10, 25 μg/mL) (Figure 4C). There were no significant variations in late apoptosis except for the highest concentration (25 μg/mL), which showed a slight but significant reduction (Figure 4D). Starvation did not lead to cell necrosis (percentage lower 1% in all conditions tested) (Figure 4B).

### 2.3. Protection by the Hexanic Octocoral Extract Against Cisplatin-Induced Ototoxicity

**Figure 5:**
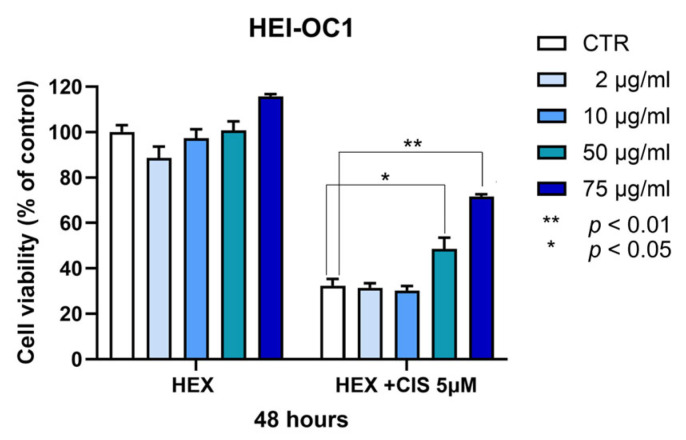
Effect of the *P. phosphorea* hexanic extract at different concentrations on HEI-OC1 cell viability after 48 h exposure, in the absence or presence of 5 μM cisplatin. Data are expressed as mean + SE (n = 4, octuplicate). Statistical evaluation was realized via ANOVA and Tukey’s post-test using GraphPad 10 software; * *p* < 0.05; ** *p* < 0.01.

Among the most ototoxic drugs, cisplatin (cis-diamminedichloroplatinum II) and other platinum salt agents may induce serious consequences in the inner ear, i.e., tinnitus and permanent damage with a high-frequency hearing impairment and outer hair cell loss [22]. In HEI-OC1 cells, cisplatin 5 μM for 48 h inhibited cell viability by about 70%. The octocoral hexanic extract slightly increased the viability of control HEI-OC1 (16%) at the maximal concentration used (75 μg/mL) (Figure 5). When the experiments were in the presence of cisplatin, the same octocoral extract gave a significant increase in cell viability not only at the maximal concentration used of 75 μg/mL (122%) but also at 50 μg/mL (57%) (Figure 5).

### 2.4. Hexane Extract of Pennatula phosphorea Exerted an Anti-Inflammatory Effect on LPS-Stimulated J774A.1 Murine Macrophages

**Figure 6:**
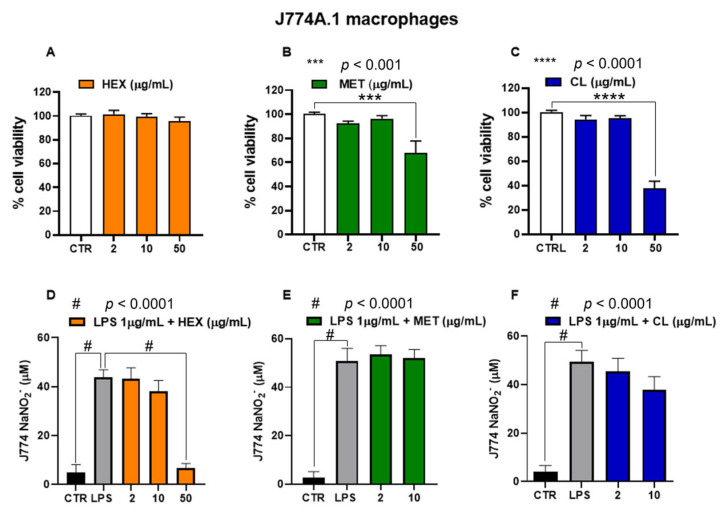
Biological activity of *Pennatula phosphorea* extracts [hexane, HEX (**A**,**D**), methanol, MET (**B**,**E**), chloroform, CL (**C**,**F**)] on murine J774A.1 macrophages. Cell viability was assessed by MTT assay in J774A.1 macrophages following a 24-hour treatment with the *Pennatula phosphorea* extracts. HEX (**A**), MET (**B**), and CL (**C**) extracts were tested at concentrations of 2, 10, and 50 μg/mL. Data are expressed as mean ± S.E.M. of four independent experiments (six replicates for each experiment). Inhibitory effect of HEX (**D**), MET (**E**), and CL (**F**) on nitrite levels was assessed in the cell medium of J774A.1 macrophages stimulated with lipopolysaccharide (LPS, 1 μg/mL) for 24 h. The extracts of *Pennatula phosphorea* were added to the cell media 30 min before LPS stimulus. Nitrite levels were measured using the Griess reagent assay. Data are expressed as mean ± SEM of three independent experiments (in triplicate). Statistical analysis was performed using ordinary one-way ANOVA followed by Tukey’s multiple comparisons test. *** *p* < 0.001; **** *p* < 0.0001; # *p* < 0.0001.

*Pennatula phosphorea* extracts (HEX, MET, and CL) were tested on J774A.1 macrophages at concentrations ranging from 2 to 50 μg/mL. In cells not treated with lipopolysaccharide (LPS), HEX extract did not exert cytotoxic effects at all concentrations used (2, 10, 50 μg/mL), while MET and CL reduced cell viability at the highest concentration of 50 μg/mL (Figure 6A–C). Thus, we investigated the effect of non-cytotoxic concentrations of HEX, MET, and CL on nitrite production in J774A.1 treated with lipopolysaccharide (LPS). 

In response to inflammatory signals, macrophages increase production of cytokines and NO. As expected, the treatment of J774A.1 macrophages with the inflammatory agent LPS (1 μg/mL for 24 h) caused a significant increase in nitrite levels, which was significantly reduced by the pre-treatment (30 min before LPS) with HEX at a concentration of 50 μg/mL (Figure 6D). As shown in Figure 6E,F, CL and MET extracts did not significantly affect nitrite production in LPS-treated J774A.1 macrophages.

### 2.5. Anti-Melanoma Activity of the Octocoral Chloroformic Extract

Cutaneous melanoma is a very aggressive skin cancer [23]. Although new therapies have significantly improved the survival of patients with advanced melanoma [24], new drugs acting on melanoma are desired to increase the armamentarium to fight this cancer when resistance or toxicity to other therapies are present. In this regard, marine-derived molecules may represent a novel interesting opportunity for treating this type of cancer.

**Figure 7:**
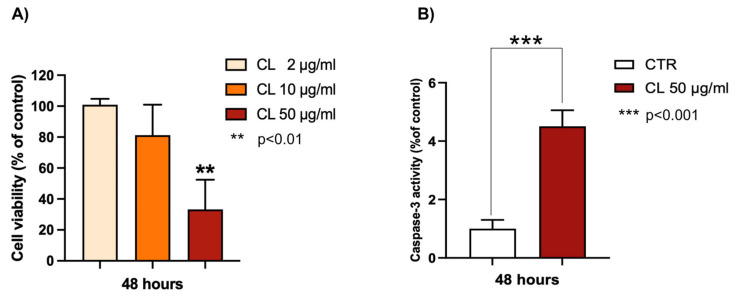
Effects of *P. phosphorea* chloroformic extract (CL) on (**A**) cell viability of melanoma A375 cells; (**B**) caspase-3 activity (50 μg/mL). Data are expressed as mean ± SE (n = 2, octuplicate (**A**); n = 2 triplicate (**B**)). ** *p* < 0.01, statistical significance between control group (untreated cells) and 50 μg CL-treated cells; *** *p* < 0.001.

The chloroformic extract from *P. phosphorea* was observed to be active against melanoma. As shown in Figure 7A, this extract is able to induce a growth inhibition of A375 melanoma cells in a concentration-dependent manner, reaching about a 70% decrease in melanoma cell viability at 75 μg/mL. To evaluate the type of cell death, the apoptotic hypothesis was investigated using caspase-3 activity. Caspases are crucial mediators of apoptosis, and caspase-3 activation has been recognized as an important target in cancer therapy [25].

As shown in Figure 7B, the chloroform extract (50 μg/mL) from *P. phosphorea* significantly increased the enzymatic activity of caspase-3 (4.5-fold increase), revealing a good pro-apoptotic ability.

### 2.6. MS-Based Molecular Networking Analysis of P. phosphorea Metabolome

With the main aim of the fast identification of compounds that may possibly underlie the bioactivities observed, an untargeted dereplication strategy of the extracts from *P. phosphorea* was employed. All extracts (hexanic, CHCl_3,_ MeOH) afforded by the modified Kupchan extraction protocol were dissolved in mass grade methanol at 1 mg/mL and analyzed by high-resolution liquid chromatography coupled with tandem mass spectrometry (LC-HRMS^2^) in the data-dependent (DDA) acquisition mode. Mass spectra were acquired both in positive and negative ion detection modes. 

The resulting raw LC-MS data, in positive and negative ESI mode, were preprocessed separately by the MZmine 2.53 software. The output files (.csv and .mgf) were exported to GNPS (Global Natural Products Social Molecular Networking), and two distinct networks were built with the feature-based molecular networking (FBMN) workflow [25,26].

Based on the similarity of the MS fragmentation patterns, FBMN provides the visual representation of compound families grouped in clusters and the annotation of known natural products reported in natural product online libraries. In brief, a node in the molecular network represents an ion detected in one or more extracts connected by edges to other nodes, which share similar MS fragmentation patterns.

**Figure 8:**
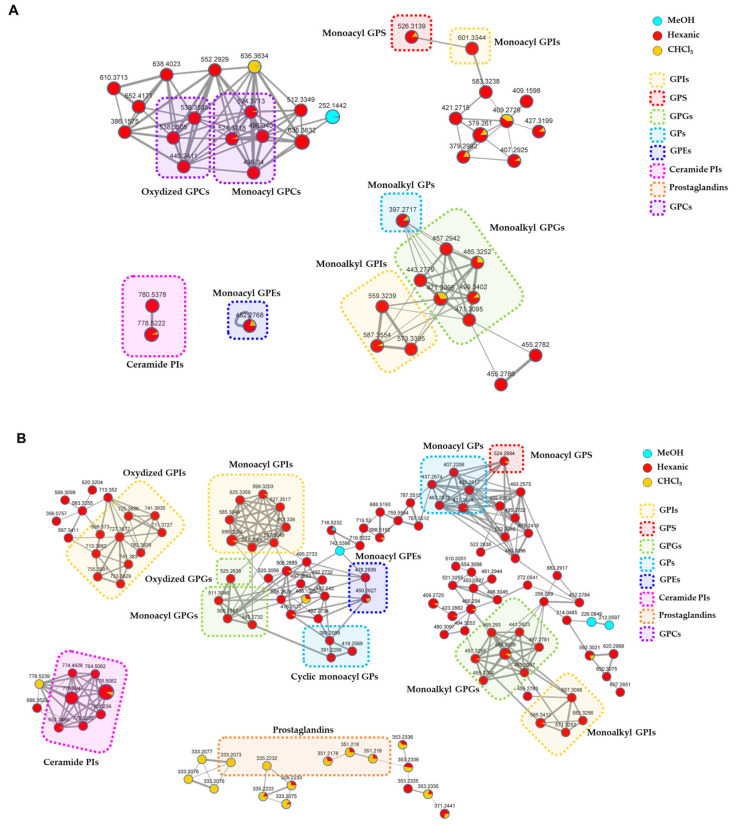
Detailed view of the annotated clusters in the molecular networks generated from LC- MS^2^ data of *P. phosphorea* extracts acquired in the positive (**A**) and negative (**B**) ion detection modes. Nodes are represented as a pie chart showing abundances of the relative precursor ion intensities in the hexanic, CHCl_3,_ and MeOH extracts. Node size reflects metabolite peak area, while edge thickness represents the cosine score similarity of the nodes. Annotated compounds are framed and mapped with different colors according to the chemical class they belong to (GPIs = Glycerophosphoinositols; GPS = glycerophosphoserines; GPGs = glycerophosphoglycerols; GPs = glycerophosphates; GPEs = glycerophosphoethanolamines; Ceramide PIs = ceramide phosphoinositols; GPCs = glycerophosphocholines).

In the networks (Figure 8, Figures S1 and S2), node size is made proportional to the area of the extracted ion chromatogram, whereas edge thickness is proportional to the cosine score, which reflects MS^2^ spectra similarity. Moreover, node colors were mapped as pie charts according to the relative precursor ion intensities in the three analyzed extracts. 

Mapping the metabolite distribution in the extracts of *P. phosphorea* enabled the prioritization of the investigation of molecular families unique to the active hexanic and CHCl_3_ extracts, thus unveiling putative molecules responsible for the observed bioactivities. Therefore, the analysis of nodes referring to compounds present—even in low concentrations—in the inactive MeOH extract was neglected. A detailed view of the clusters grouping annotated compounds in the molecular networks generated from LC-MS^2^ data from *P. phosphorea* extracts is shown in Figure 8. The full molecular networks are displayed in Figures S1 and S2.

The relevant chemical classes of the metabolites are highlighted in frames with different colors. The thorough examination of the molecular networks and the following investigation of the MS^2^ spectra led to the putative annotation of about 50 compounds, which were assigned to eight chemical classes, namely glycerophosphoinositols (GPI), glycerophosphoglycerols (GPG), glycerolphosphocholines (GPC), glycerophosphoserines (GPS), glycerophosphoetanolammines (GPE), glycerophosphates (GP), ceramide phosphoinositols (IPC), and prostaglandins. Overall, the majority of the clusters exclusive or more abundant in the hexanic extract have been shown to be related to phospholipids and phosphosphingolipids, while a few clusters grouping prostaglandins (PG) were mostly detected in chloroformic extract.

#### 2.6.1. Glycerophospholipids

Glycerophosphoinositols (GPIs) are a class of glycerophospholipids whose structure consists of a glycerol backbone with one free hydroxyl function, at either the *sn*-1 or the *sn*-2 position, linked to a phosphoinositol molecule. A long acyl (monoacyl GPIs) or alkyl/alkenyl chain (monoalkyl/alkenyl GPIs), varying in length and degree of desaturation, usually esterifies the *sn*-2 position of the glycerol unit. 

**Figure 9:**
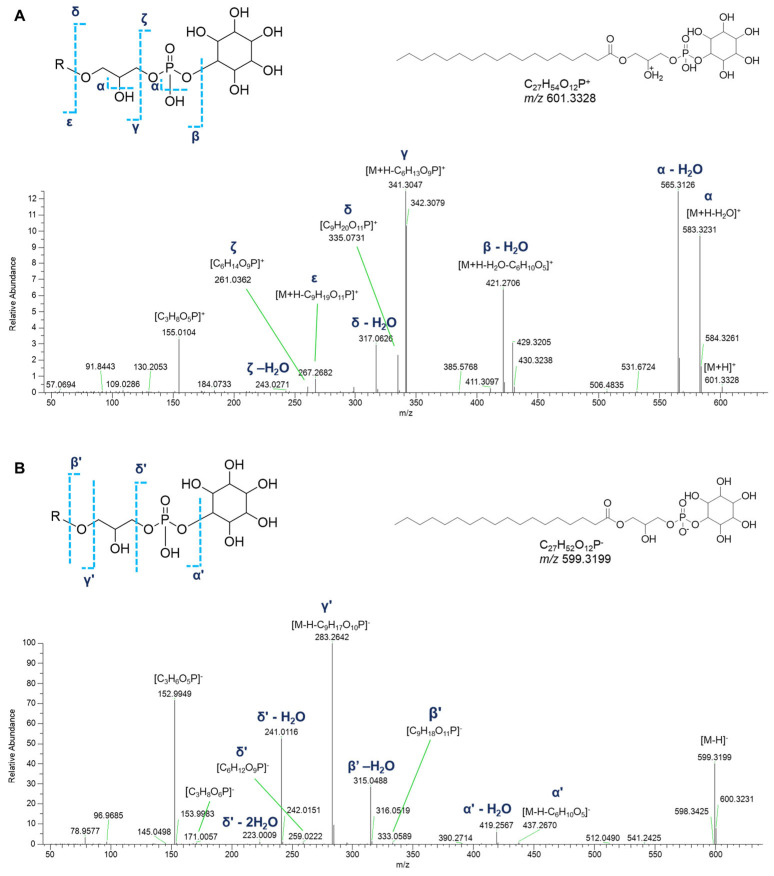
HR ESI-MS^2^ spectra of the [M+H]^+^ (**A**) and [M−H]^−^ (**B**) ions of a representative lyso-phosphatidylinositol (GPI) from *P. phosphorea* (LPI 18:0).

Monoacyl and monoalkyl GPIs were detected in both positive and negative ion detection modes (Table 1). Besides the neutral losses of water molecules from the phosphate group or the glycerol moiety, the fragment ion derived from inositol phosphate loss [M+H-C_6_H_13_O_9_P]^+^ dominated the [M+H]^+^ spectra of GPI lipids. The glycerylphosphorylinositol ion at *m*/*z* 335.0738 ([C_9_H_20_O_11_P]^+^) and the phosphorylinositol ion at *m*/*z* 261.0370 ([C_6_H_14_O_9_P]^+^), and their respective ions deriving from neutral water losses, represent the diagnostic fragment peaks for the recognition of GPI lipids. Moreover, ions arising from the loss of the glycerylphosphorylinositol [M+H-C_9_H_19_O_11_P]^+^ and the fragment ions arising from the sequential losses of water and the inositol residue ([M+H-H_2_O-C_6_H_10_O_5_]^+^) were useful to clearly identify the fatty acid chains (Figure 9A). 

Acquisition of negative HR ESI-MS spectra allows the detection of other phospholipids having the same glycerylphosphorylinositol moiety. In the [M−H]^-^ spectra of GPI lipids, the structurally informative peaks are (a) the fragment ion at *m*/*z* 259.0224 ([C_6_H_12_O_9_P]^−^) and the related mono- and di-dehydrated ions, representative of the phosphorylinositol moiety, and (b) the glycerylphosphorylinositol fragment ion at *m*/*z* 333.0592 ([C_9_H_18_O_11_P]^−^) resulting from the loss of the fatty acid group as ketene and the related dehydrated ion at *m*/*z* 315.0487. Further information to infer the fatty acyl substituent was obtained thanks to the ions generated by loss of the glycerylphosphorylinositol group ([M−H-C_9_H_17_O_10_P]^−^). Moreover, the presence of the fragment ion at *m*/*z* 171.0064 ([C_3_H_8_O_6_P]^−^), corresponding to the glycerol phosphate, together with its dehydrated form at *m*/*z* 152.9958, are common peaks in monoglycerophospholipid mass spectra. Notably, ten oxidized glycerylphosphorylinositols could be detected in the molecular network, bearing hydroxy and/or oxo fatty acyl substituents (Table 1, Figure 9B).

Lysoglycerophosphoglycerols (LPGs) are a class of monoglycerophospholipids, composed of a glycerol unit linked to a phosphoglycerol molecule with a long acyl (monoacyl glycerophosphoglycerols (GPGs)) or alkyl/alkenyl chain (monoalkyl/alkenyl GPGs). Overall, a group of monoacyl GPGs were dereplicated in negative ion detection mode, while LPG esterified with an alkyl/alkenyl group were ionized in both positive and negative acquisition mode (Table 2). 

**Figure 10:**
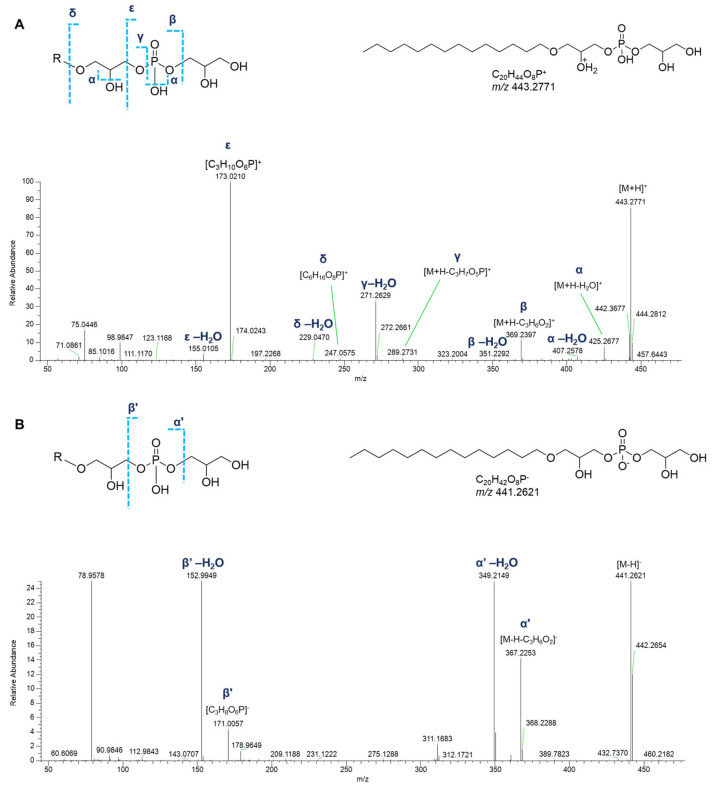
HR ESI-MS^2^ spectra of the [M+H]^+^ (**A**) and [M−H]^−^ (**B**) ions of a representative monoalkyl glycerophosphoglycerol (GPG) from *P. phosphorea*, annotated (LPG O-14:0).

In detail, apart from the fragment ions corresponding to the neutral losses of water molecules from the phosphate group or the glycerol moiety, the product ion spectra of the [M+H]^+^ adducts of monoalkyl GPGs displayed the informative fragment ions of the glycerylphosphorylglycerol unit, i.e., the daughter ions at *m*/*z* 247.0577 ([C_6_H_16_O_8_P]^+^) and at *m*/*z* 173.0210 ([C_3_H_10_O_6_P]^+^) and the ions produced by their respective neutral water losses. The fragment ions generated by the elimination of the glycerophosphate moiety ([M+H-C_3_H_7_O_5_P]^+^) and of the glycerol group ([M+H-C_3_H_6_O_2_]^+^), including their dehydrated forms, allowed to shed light on the composition of the alkyl chain (Figure 10A). 

Only few peaks were evident in the acquisition of negative HR ESI-MS spectra of monoalkyl GPGs: (a) the fragment originated from the neutral loss of the glycerol group ([M-H-C_3_H_6_O_2_]^−^), (b) the fragment ion at *m*/*z* 171.0064 ([C_3_H_8_O_6_P]^−^), and the ions resulting from further water losses (Figure 10B).

In contrast to the negative tandem mass spectra of monoalkyl LPGs, the fragmentation pattern of the LPGs binding fatty acyl chains was more informative. In addition to the already described fragment ions, the product ion spectra displayed the daughter ions at *m*/*z* 245.0432 ([C_6_H_14_O_8_P]^−^) and the ions originated from an additional neutral water loss, which are indicative of the glycerylphosphorylglycerol backbone. The fragment ions generated by the elimination of the glycerylphosphorylglycerol moiety ([M−H-C_6_H_13_O_7_P]^−^) dominated the spectra and allowed for fatty acid chain assignment (Figure S3). 

**Figure 11:**
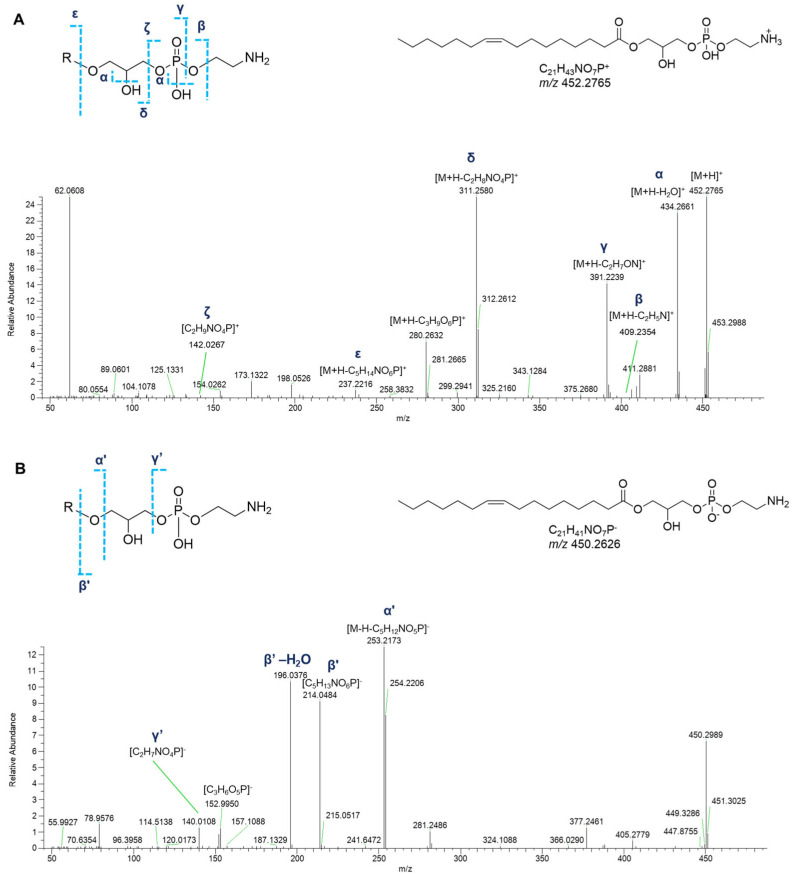
HR ESI-MS^2^ spectra of the [M+H]^+^ (**A**) and [M−H]^−^ (**B**) ions of a representative monoacyl glycerophosphoethanolamine (GPEs) from *P. phosphorea* (LPE 16:1).

Dereplication of *P. phosphorea* extracts allowed for the identification of a group of monoacyl glycerophosphoethanolamines (GPEs, Table 3). Overall, the product ion spectra were in line with the fragmentation patterns of GPEs previously described [27]. The abundant fragments in the tandem mass spectra of the [M+H]^+^ ions of lyso-PEs encompass the daughter ions resulting from water loss from the precursor ion and the [M+H-C_2_H_8_NO_4_P]^+^ peak derived from the elimination of the phosphoethanolamine unit via the phosphoester bond cleavage (Figure 11A). Additionally, ions resulting from rearrangement reactions included (a) [M+H-C_2_H_5_N]^+^ arising from loss of aziridine (C_2_H_5_N), (b) [M+H-C_2_H_7_NO]^+^ following loss of ethanolamine (C_2_H_7_NO), and (c) [M+H-C_3_H_9_O_6_P]^+^ from internal loss of glycerophosphoric acid (C_3_H_9_O_6_P). Less informative were the MS^2^ spectra of GPEs in negative ion mode, which showed the fragment peak [M-H-C_5_H_12_NO_5_P]^−^, coming from the loss of glycerophosphoethanolamine, as the dominant ion of the spectra. Furthermore, ions with lower intensity, i.e., the ions at *m*/*z* 214.0486 ([C_5_H_13_NO_6_P]^−^) (together with its dehydrated form) and at *m*/*z* 140.0118 ([C_2_H_7_NO_4_P]^−^) clearly confirmed the phosphoglyceroethanolamine backbone. Moreover, the ion at *m*/*z* 152.9958 is consistent with a monoglycerophospholipid structure (Figure 11B). 

**Figure 12:**
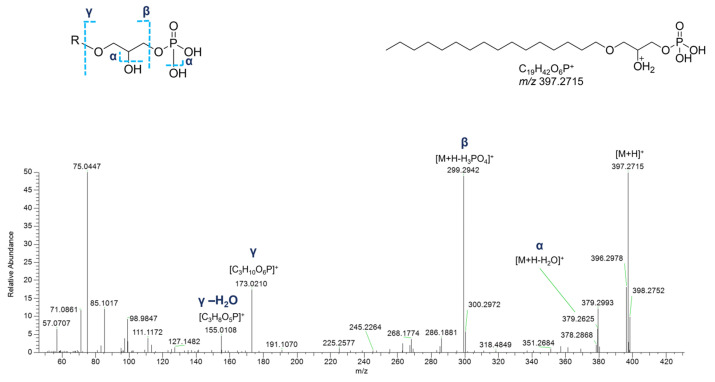
HR ESI-MS^2^ spectrum of the [M+H]^+^ ion of a putative monoalkyl glycerophosphate (GPA) from *P. phosphorea* (LPA O-16:0).

In the cluster where the majority of glycerophospholipids were grouped, several glycerophosphates (GPs), including cyclic monoacyl GPs, were detected (Table 4). Interestingly, monoalkyl GPs were only identified in positive ESI-MS HR spectra, while monoacyl GPs were only detected in the negative ones. Typical fragmentations of tandem mass spectra of the [M+H]^+^ ions of monoalkyl GPs comprised (a) the [M+H-H_2_O]^+^ fragment ion, (b) the [M+H-97.9769]^+^ peak resulting from loss of phosphoric acid (H_3_PO_4_), and (c) the glycerophosphosphoric acid fragment ([C_3_H_10_O_6_P]^+^, Figure 12). 

**Figure 13:**
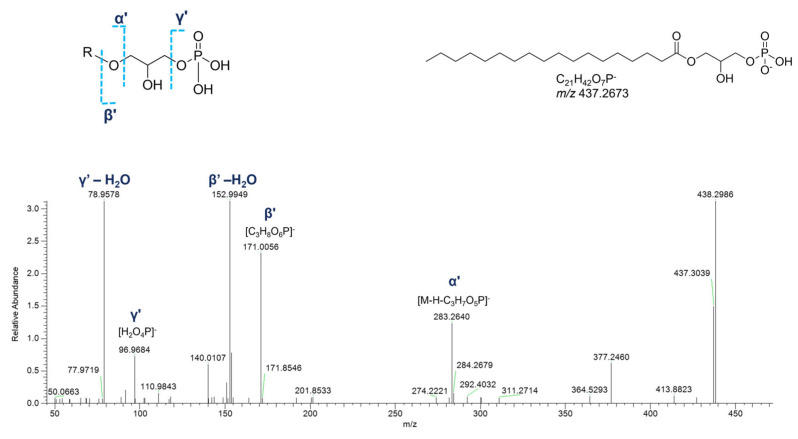
HR ESI-MS^2^ spectrum of the [M−H]^−^ ion of a representative monoacyl glycerophosphate (GPA) from *P. phosphorea* (LPA 18:0).

In the negative polarity mode, spectra of monoacyl GPs, in addition to the fragment ions referred to the phosphoric acid at *m*/*z* 96.9696 ([H_2_O_4_P]^−^) and the glycerophosphoric acid at *m*/*z* 171.0064 ([C_3_H_8_O_6_P]^−^), the daughter peak [M-H-C_3_H_7_O_5_P]^−^ provided information about the fatty acid chain (Figure 13). Much simpler is the negative ion mass spectra of cyclic monoacyl GPs, showing the cyclic glycerophosphoric ion at *m*/*z* 152.9958 ([C_3_H_6_O_5_P]^−^) and the fragment resulting from the neutral loss of cyclic glycerophosphoric acid ([M-H-C_3_H_5_O_4_P]^−^) (Figure S4).

One monoacyl glycerophosphoserine (LPS 18:0) was identified in the extract, both in positive and in negative ion detection modes (Table 5). LPSs are serine derivatives in which the hydroxyl side chain of the amino acid is linked to the glycerophosphoric group. In the positive HR ESI-MS^2^ spectrum, the MS^2^ ions at *m*/*z* 260.0530 ([C_6_H_15_NO_8_P]^+^) and at *m*/*z* 106.0503 ([C_3_H_8_NO_3_]^+^), together with their respective neutral water losses, clearly suggest a glycerophosphoserine structure (Figure S5). In addition, the ion at *m*/*z* 106.0499 ([C_3_H_8_NO_3_]^+^), corresponding to serine, along with the ion at *m*/*z* 88.0393 arising from further loss of H_2_O, were seen in the spectrum. Furthermore, (a) the fragment arising from phosphoserine ([M+H-C_3_H_8_NO_6_P]^+^) loss, (b) the glycerophosphoserine ion ([C_6_H_15_NO_8_P]^+^), and (c) the fragment deriving from removal of the terminal serine at *m*/*z* 421.2714 ([M+H-C_3_H_7_NO_3_]^+^), allowed for the structural elucidation of the acyl chain. The MS fragmentation pattern of the [M–H]^−^ ion showed few peaks, such as (a) the fragment at *m*/*z* 437.2674 ([M-H-C_3_H_5_NO_2_]^−^), generated after loss of the serine residue from the precursor ion, with its cognate dehydrated ion, and (b) the carboxylate anion at *m*/*z* 283.2643 ([M-H-C_6_H_12_NO_7_P]^−^), arising from glycerophosphoserine loss. Again, the glycerophosphate ion at *m*/*z* 171.0064 ([C_3_H_8_O_6_P]^−^) was observed in the spectrum (Figure S5).

Two putative monoacyl glycerophosphocholines (GPCs) and two oxidized glycerophosphocholines were annotated through the analysis of fragmentation patterns of their precursor ions as protonated adducts (Table 6). The diagnostic signals displayed in the spectra were (a) the phosphocholine ion at *m*/*z* 184.0733 ([C_5_H_15_NO_4_P]^+^), (b) the ion at *m*/*z* 104.1070 ([C_5_H_14_NO]^+^) indicative of the choline headgroup, and (c) the glycerylphosphorylcholine daughter ion at *m*/*z* 258.1101 ([C_8_H_21_NO_6_P]^+^) together with the cognate ion resulting from the water loss. Moreover, ions generated from the loss of the phosphocholine head group ([M+H-183.0655]^+^) disclosed the linked fatty acid chain (Figure S6).

#### 2.6.2. Phosphosphingolipids

**Figure 14:**
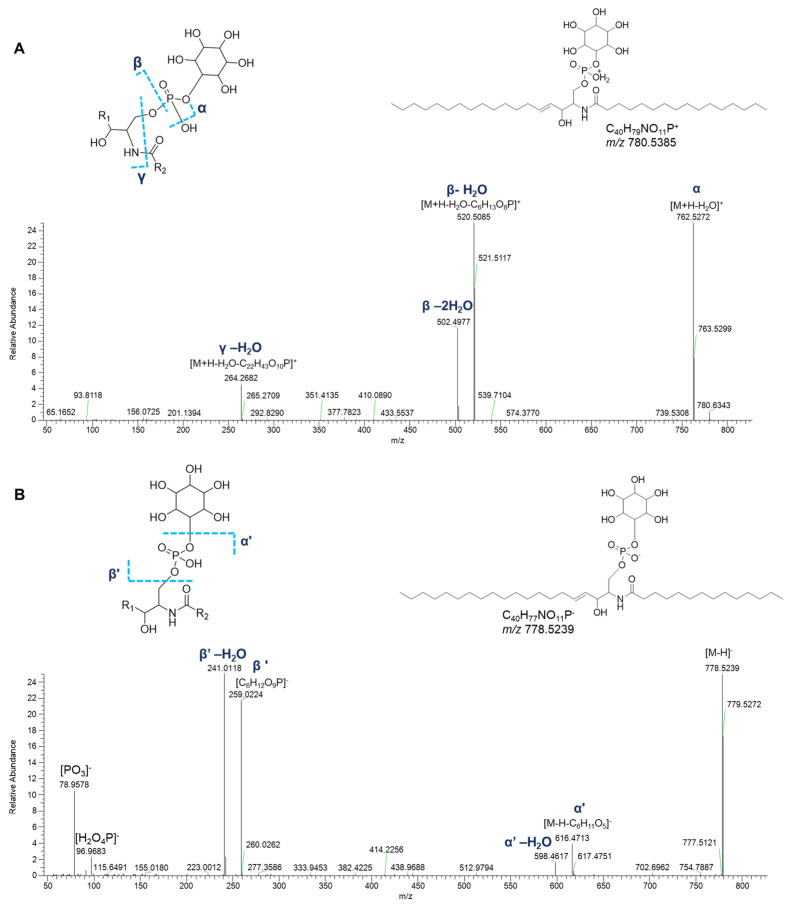
HR ESI-MS^2^ spectra of the [M+H]^+^ (**A**) and [M−H]^−^ (**B**) ions of a representative ceramide phosphoinositol (IPC) from *P. phosphorea* (IPC (d18:1/16:0)).

Ceramide phosphoinositols (IPCs) were nicely grouped in a single cluster in both positive- and negative-ion mode networks. IPCs are formed by a ceramide moiety, linked to an inositol phosphate residue via the C1 OH group of ceramides. Analysis of [M+H]^+^ and [M–H]^−^ adducts of IPC species led to the putative annotation of 6 compounds (Table 7). The positive MS^2^ spectra are dominated by the ion ([M+H-H_2_O-C_6_H_13_O_8_P]^+^) generated from the consecutive losses of the inositol monophosphate and a water molecule (Figure 14). Additionally, the fragments derived by the elimination of another water molecule from the last-mentioned ion and by the elimination of water from the parent ions were displayed in Figure 14. The mechanism of these rearrangements was previously proposed by Hsu et al. in 2007 [28]. The spectra also contained the ions corresponding to the sphingosine or sphingodienine residue, i.e., [M+H-H_2_O-C_6_H_13_O_9_P-C_16_H_30_O]^+^, arising from losses of water, inositol monophosphate, and the fatty acyl ketene. In the ESI-MS profile of the [M–H]^−^ adduct of IPCs isolated from *P. phosphorea*, the prominent ions at *m*/*z* 259.0224 ([C_6_H_12_O_9_P]^−^) and at *m*/*z* 241.0119 referred to an inositol monophosphate anion and to an inositol-1,2-cyclic phosphate anion, respectively [28]. Additional characteristic fragments of inositol phosphorylceramides are the ions [M-H-C_6_H_11_O_5_]^-^ and the related neutral water losses, which are indicative of inositol loss (Figure 14). 

#### 2.6.3. Prostaglandins (PGs)

Five nodes displayed in three different clusters of the molecular network generated with ESI-negative data were putatively annotated as prostaglandins (Figure 8). Prostaglandins (PGs) are fatty acid derivatives with a distinctive twenty-carbon skeleton, featuring a cyclopentanone nucleus bearing oxygen atoms in specific positions. Two side chains are connected to the central five-membered ring, whose features (chain length, degree of unsaturation, functional groups) influence the reactivity and the biological properties [29].

PGs are classified according to the number of double bonds within the prostaglandin molecule and the fatty acid from which they are synthesized. Accordingly, prostaglandins of the 1 series have one double bond and are derived from dihomo-γ-linolenic acid, PGs of the 2 series feature two double bonds and derive from arachidonic acid, and those of the 3 series possess three double bonds and derive from eicosapentaenoic acid. In addition, prostaglandins are further grouped in series, often indicated by letters (e.g., A, B, C, D, E, F, G, H), in accordance with the functional substitutions in the cyclopentanone core.

Fragmentation patterns of compounds belonging to the prostaglandin family have been clearly described, showing intense product ions resulting from consecutive losses of H_2_O and carbon dioxide, together with ions generated by the α-hydroxy cleavage of the C-14 and C-15 bond in the vinyl position and fragments produced by the cleavage of the carboxyl group with further losses of H_2_O, CO_2_ [30,31,32,33].

**Figure 15:**
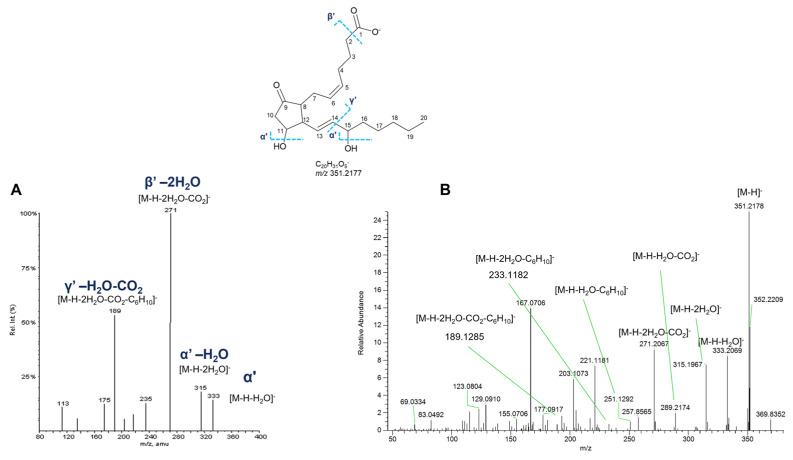
HR ESI-MS^2^ spectra of the [M−H]^−^ ion of PGE_2_ (*m*/*z* 351.2177) retrieved from LIPIDMAPS (**A**) and [M−H]^−^ ion at *m*/*z* 351.2180 (*R*_t_ = 19.0 min) from *P. phosphorea* (**B**).

Experimental MS^2^ spectra of putative PGs from *P. phosphorea* are in agreement with the above-described fragmentation patterns, as displayed in Figure 15, showing similar product ion spectra compared with the already reported ones. However, it is impossible to distinguish unambiguously isomers with the same parent mass, i.e., the PGE_2_ and PGD_2_, by mass spectrometry, as their MS^2^ spectra are virtually identical. For this reason, putatively identified compounds are referred to by the LIPID MAPS shorthand notation at the species level (sum composition) (Table 8) [34]. 

## 3. Discussion

The sea pen *P. phosphorea* is a coastal species found on sandy or muddy sediments from 15 to 100 meters depth, widespread in the Mediterranean Sea, the North-East Atlantic Ocean, and the North Sea [35]. Thus far, there have been few reports about the chemical composition and biological potential of its extracts. It should be mentioned that the investigation of a specimen of *P. phosphorea* collected from the Gullmar fjord (Bohuslan, Sweden) showed the presence of a pool of carotenoids (canthaxanthin, isozeaxanthin, zeaxanthin, astaxanthin, and astacene) [36], while a preliminary study on the chemical defenses of two octocorals demonstrated that dichloromethane and methanol extracts of *P. phosphorea* exhibit narcotic and anorectic properties, and the latter was also seen to act as a feeding deterrent using the Dover sole, *Solea solea*, as the test animal [37]. In this work, crude extracts of *P. phosphorea*, obtained with a modified Kupchan extraction protocol, were subjected to a panel of pharmacological assays, showing promising biological activities for the hexanic and chloroformic extracts. 

A protective effect of the hexanic extract was revealed in muscle cells damaged to reproduce sarcopenia, a complex multifactorial condition characterized by the impairment of muscle components and functionality, influenced by factors like inactivity, malnutrition, hormonal imbalances, insulin resistance, and drugs [38]. Glucocorticoids, such as dexamethasone, exacerbate muscle atrophy by inhibiting protein synthesis and promoting protein degradation [39]. 

All extracts were tested in an in vitro model of sarcopenia obtained by treating C2C12, mouse skeletal myoblasts, with dexamethasone. For the hexanic extract, a starvation condition was additionally evaluated to mimic a state of malnutrition. HEX improved myoblast viability in a concentration-dependent manner, as well as it favored the differentiation of cells into myotubes. Accordingly, it prevents early apoptotic processes.

The anti-inflammatory effect of the hexanic extract from *P. phosphorea* was also confirmed in murine macrophages. Specifically, we evaluated the effect of hexanic, methanolic, and chloroformic extracts on nitric oxide (NO) production in macrophages activated with LPS. Stimulation of macrophages with LPS causes a significant increase in NO, which is reduced by the treatment with hexanic extract at non-cytotoxic concentrations. Methanolic and chloroformic extracts of *P. phosphorea* did not affect the production of nitric oxide in murine macrophages, suggesting the potential anti-inflammatory effect only for the hexanic extract. Extracts of *P. phosphorea* were also tested as protective agents against cisplatin-induced ototoxicity, revealing the octocoral hexanic extract to give a significant increase in cell viability in HEI-OC1 cisplatin-treated cells.

Although lacking efficacy in the previously reported assays, the chloroform extract was active in reducing melanoma cell viability, being able to induce a growth inhibition of A375 melanoma cells in a concentration-dependent manner.

With the main purpose of identifying compounds that may be responsible for the observed bioactivities, extracts of *P. phosphorea* were chemically profiled using high-resolution LC-MS^2^ and a molecular networking approach, in its feature-based molecular networking variant (FBMN), followed by thorough manual analysis of HR ESI-MS^2^ data. 

Molecular networking (MN) is a powerful MS-based computational tool that has become a key natural product research method. It enables the organization and visualization of large MS^2^ datasets, i.e., complex organic mixtures, comparing compound similarity in their fragmentation spectra and enabling the rapid identification of known natural products (dereplication) and their unknown analogs, as well as the identification of completely new NPs [40,41]. 

Careful dereplication of the molecular networks generated from positive and negative ion modes of LC-MS^2^ data from *P. phosphorea* extracts resulted in the annotation of different classes of glycerophospholipids, phosphosphingolipids, and prostaglandins (Figure 8), which, to the best of our knowledge, have been reported herein for the first time from sea pens. In addition, the monoalkyl and the oxidized glycerophospholipids (Table 1, Table 2, Table 4 and Table 6), as well as all the ceramide phosphoinositols (Table 7) detected in *P. phosphorea,* have not been reported in the LIPID MAPS database so far and, therefore, are putative novel lipid variants (Tables S2 and S3).

Among them, glycerophospholipids, including glycerophosphoinositols (GPI), glycerophosphoglycerols (GPGs), glycerophosphoethanolamines (GPEs), glycerophosphates (GPs), cyclic GPs, glycerophosphoserines (GPSs), and glycerophosphocholines (GPCs), as well as the sole phosphosphingolipid class of ceramide phosphoinositols (IPCs), were shown to be exclusive or more abundant in the hexanic extract, while a group of prostaglandins was mostly eluted in chloroformic extract (Figure 8).

The majority of the putatively identified glycerophospholipids were lysophospholipids, i.e., phospholipids lacking a fatty acid chain from either the *sn*-1 or *sn*-2 position. Monoacylated/monoalkylated glycerophospholipids are structural constituents of cell membranes and biosignaling molecules involved in many cellular processes in eukaryotes and bacteria, including marine species. 

Corals are recognized to be rich in lipids, which play a key role in maintaining their health and metabolism [42]. Identified within phospholipid compositions of soft corals were GPCs, GPEs, GPSs, GPIs, and GPs, while GPGs are known to be produced by photosynthesizing microalgae (zooxanthellae), common endosymbionts of marine organisms such as coral polyps [42,43]. At present, a total lipidomic profile has only been characterized in a few soft corals, none belonging to the order Pennatulacea [44,45,46,47]. Interestingly, glycerophospholipids from marine origin have been shown to exert potent cytoprotective potential [48]. In detail, lysophospholipids extracted from the sea cucumber *Holothuria atra* exerted potent hepatoprotective effects in vitro [48]. Relevant recent work reports that marine phospholipids exhibit cardioprotective effects due to their anti-inflammatory, anti-thrombotic, and immunomodulatory properties [49].

Among phosphosphingolipids, a group of six ceramide phosphoinositols (IPCs) were putatively annotated in *P. phosphorea* extracts. Inositolphosphoryl ceramides are commonly occurring in fungi and protista kingdoms but have never been reported in mammals [50]. Only a few examples of IPCs were reported from marine species: CJP1 isolated and structurally characterized from the feather star *Anneissia japonica* (former *Comanthus japonica*) [51], a new compound with inositol phosphoceramide structure from red algae *Gracilaria verrucosa* [50], and zeamide, the first example of a new class of glycosylinositol phosphorylceramides, in which the inositol is glycosylated by a D-arabinose isolated from the Caribbean sponge *Svenzea zeai* [52]. Little is known about the subcellular distribution and biological functions of IPCs. The only information was reported from the IPCs of the yeast *Saccharomyces cerevisiae*, which have been shown to be substantial components of biological membranes and necessary factors for yeast growth, viability, and resistance to stress [50]. 

The anti-inflammatory effect of the hexanic extract could be at least partially due to the presence of glycerophospholipids and phosphosphingolipids, demonstrated to modulate cellular inflammation pathways (Table 9). Both classes of compounds play a key role in maintaining the structural integrity of cell membranes, essential for proper muscle contraction and function. Additionally, these lipids modulate signaling pathways like NF-κB, MAPK, or PI3K/Akt, which may help reduce inflammation associated with muscle degeneration [53,54]. In particular, dysregulation of glycerophospholipid metabolism has been linked to age-related muscle loss [55]. On the other hand, phosphosphingolipids, including ceramide phosphoinositols, regulate apoptotic signaling through pathways involving caspase activation and mitochondrial integrity, offering protection against cell death and inflammation (Table 9) [56]. Moreover, glycerophospholipids are essential components of mitochondrial membranes and play a significant role in enhancing mitochondrial activity [57]. Dysfunctional mitochondria are now well acknowledged as a factor in age-associated muscle atrophy and functional decline [58]. The role of mitochondrial activity in muscle health is supported by several studies on natural compounds [54,59]. 

The above anti-inflammatory and anti-apoptotic mechanisms by the phospholipid molecules in the hexanic extract (Table 9) may also contribute to its otoprotective effect observed against cisplatin-induced toxicity. In fact, the mechanism underlying the cisplatin ototoxic effect is complex, encompassing oxidative stress but also inflammatory mediators and different cell death pathways, among which apoptosis [60]. Noteworthy, ceramide-1-phosphate (C1P), a phosphorylated form of ceramide, showed protective effects in the cisplatin ototoxicity, successfully inhibiting cisplatin-induced cochlear outer hair cell death [61].

**Table 9:** Mechanism of action reported for lipid classes from *P. phosphorea*. Molecules detected in *P. phosphorea* extracts are indicated in bold.

Compounds	Bioactivity	Mechanism of Action	Reference
**LPC 16:0**, LPC O-16:0, LPC O-18:1, **LPC 18:0**, LPC O-18:0, LPC O-19:0	cytoprotective in macrophages	H_2_O_2_-induced apoptosis inhibition	[48]
**LPC 16:0**, PC 18:2/18:2, PC 16:0/16:0, PC 16:0/18:2	anti-inflammatory in Caco-2 cells	inhibition of TNF-α-induced NF-κB activation	[49]
ceramide-1-phosphate	protection against cisplatin ototoxicity	activation of the Akt and MAPK pathway	[62]
**LPI 18:0**	neuroprotection in microglial cells	reduction of LPS-induced NO production suppression of ROS generation and cytokine release suppression of microglial phagocytosis	[62]
**LPI 16:0**, LPC 14:0, **LPC 16:0**	neuroprotection against ischemia and excitotoxicity	putative activators of 2P-domain K^+^ channels	[63]
**LPG 16:0**	anti-inflammatory in phagocytes	inhibition of formyl peptide receptor like-1 (FPRL1)	[64]
**LPG 18:1**, LPC 18:1, **LPE 18:1**, **LPI 18:1**, LPS 18:1	anti-inflammatory in HT-29 cells	decreased IL-8 secretion	[65]
LPE 20:4	anti-inflammatory in macrophages and carrageenan-induced paw edema	inhibition of iNOS, COX-2, IL-1β, IL-6, and IL-12 expression	[66]
**LPA 18:0**, **LPA 18:1**	reduction in LPS-caused organ injury	activation of LPA G-protein-coupled receptor and PPAR-γ	[67]
CPA 18:1	protection of neuroblastoma neuro2A cells from hypoxia-induced Apoptosis	LPA2 activation	[68]
Δ^7^-PGA_1_, Δ^12^-PGJ_2_, PGJ_2_, PGA_2_, PGD_2_, PGA_1_, PGE_2_, PGE_1_	anti-tumor against ovarian cancer cells	cell cycle arrest, apoptosis, myc inhibition	[69]

Five nodes only appearing in the molecular network generated with ESI-negative data were putatively annotated as prostaglandins (Figure 8). PGs have been proven to play essential roles in mammals, being implicated in many physiological and pathological processes such as regulating vascular tone, signaling, reproduction, and mediating inflammatory and immune responses [29]. In addition, recent studies have evidenced that PGs participated in the tumor process, showing different roles according to many factors, including the target tissue, the concentration and prostaglandin subtype, and the signaling pathways in play [70]. Indeed, COX-derived eicosanoids, in particular PGE_2_, are recognized mediators involved in carcinogenesis and in cancer progression, producing cancer cell proliferation, angiogenesis, and resistance to apoptosis and metastasization [71]. Confirming this role in melanoma, selective inhibition of COX-2 activity was shown to reduce cellular proliferation and invasiveness [72]. On the other hand, PGD_2_ type prostaglandins are protective agents inhibiting tumor progression, either as a primary tumor or as metastases (see also Table 9). [70]

PGs are also essential molecules in marine organisms, acting in reproduction, the regulation of oxygenation and osmotic pressure, and in defense mechanisms and communication [73,74,75]. It has been reported that marine invertebrates, especially soft coral, contain a wide variety of prostaglandins, both conventional types (i.e., PGA_2_, PGE_2_, PGD_2_, PGF2α) and specific PGs not occurring in mammals, exhibiting mainly anti-cancer but also anti-inflammatory and anti-viral activities [29,74]. Marine prostaglandins were first identified in the Caribbean gorgonian *Plexaura homomalla,* whose extracts contain a large mixture of PGs [76]. Prostaglandin A_2_ type compounds isolated from the aforementioned octocoral were investigated, revealing cytotoxic properties against breast (MDA-MB-231) and lung (A549) cancer cell lines [77]. Furthermore, octocorals belonging to the genus *Clavularia* are a rich source of cytotoxic and anti-proliferative prostanoids against various cancer cell lines, including A549 (human lung adenocarcinoma), HT-29 (human colon adenocarcinoma), MOLT-4 (human T lymphocyte leukemia) [7,78,79,80]. In accordance, the presence of PGs in the *P. phosphorea* chloroform extract could explain its activity in reducing melanoma cell viability. 

Many additional nodes in the MNs could not be annotated as their *m*/*z* and putative molecular formulas were not present in databases used for dereplication, implying plenty of new chemistry in the extracts that could potentially contribute to the tested bioactivities (Figure 8, Figures S1 and S2). In conclusion, the extracts of the sea pen *P. phosphorea* have proven to be a promising source of valuable compounds, with remarkable bioactivities. This result prompts further investigations, eventually leading to the isolation of individual bioactive chemical entities.

## 4. Materials and Methods

### 4.1. Collection and Extraction 

Specimens of the octocoral *P. phosphorea* (Anthozoa: Octocorallia), investigated in this study, were collected along the continental shelf off Portopalo di Capo Passero (Southern Sicily Island, Central Mediterranean Sea). Immediately after collection, the sea pen was stored at −20 °C until extraction. A modified Kupchan extraction scheme was performed. In detail, the frozen specimens of *P. phosphorea* were cut into small pieces and extracted at room temperature three times with MeOH under stirring for 2 h each. After this step, the MeOH extract was concentrated under vacuum at the rotary evaporator to 50 mL, to which 5 mL of dH_2_O was added. The obtained extract was partitioned with 50 mL of hexane. The hexane phase was then dried, yielding 70.7 mg, while the MeOH/H_2_O phase was further extracted with 50 mL of CHCl_3_, after the addition of 30 mL of dH_2_O. The resulting CHCl_3_ layer was dried under vacuum, affording 82.8 mg. Hence, the MeOH/H_2_O phase was extracted with 50 mL of EtOAc, with the addition of 50 mL dH_2_O. The EtOAc extract weighed 7.2 mg after drying. Finally, the MeOH/H_2_O phase was flushed through an RP-18 SPE cartridge, washed with dH_2_O (5 mL), removing salts, and eluted with 50 mL of MeOH. The dried MeOH extract (624.3 mg) was then combined with EtOAc extract, given the poor yield of the latter. The resulting Kupchan extracts (MeOH, CHCl_3_, and hexanic extracts) were dissolved in DMSO for bioactivity assays and in MeOH for tandem mass spectrometry molecular networking analyses.

### 4.2. Cell Cultures

C2C12 mouse skeletal myoblasts were purchased from American Type Culture Collection (Manassas, VA, USA), cultured in Dulbecco’s Modified Eagle Medium (DMEM) supplemented with 10% fetal bovine serum (FBS), 100 U/mL penicillin, 100 g/mL streptomycin, 2 mM L-glutamine (Life Technologies Italia, Milan, Italy), and maintained at 37 °C in a humidified atmosphere with 5% CO_2_. C2C12 myoblasts were cultured until reaching 80% confluency.

HEI-OC1 (House Ear Institute-Organ of Corti 1) cells, kindly gifted by Prof. F. Kalinec (UCLA, University of California, Los Angeles), were maintained in high-glucose Dulbecco’s modified Eagle’s medium (DMEM) (Euroclone, Pero, Italy) supplemented with 10% fetal bovine serum (FBS) and 2.5 μg/mL amphotericin B (Sigma/Merck, Germany), at 33 °C in a humidified incubator with 10% CO_2_, as described by Kalinec et al. (2016) [81].

J774A.1 macrophages (ATCC, from LGC Standards, Milan, Italy) were routinely maintained at 37 °C in a humidified incubator with 5% CO_2_, and were cultured in Dulbecco’s modified Eagle’s medium (DMEM, Lonza Group) supplemented with 10% FBS, 100 U/mL penicillin and 100 μg/L streptomycin, 2 mM L-glutamine, 20 mM Hepes (4-(2-hydroxyethyl)-1-piperazineethanesulphonic acid), and 1 mM sodium pyruvate. The medium was changed every 48 h in conformity with the manufacturer’s protocols, and cell viability was evaluated by trypan blue exclusion. A375 melanoma cells, kindly gifted by Dr. L. Poliseno (Institute of Clinical Physiology CNR, Pisa, Italy), were cultured in DMEM (Euroclone, Italy) supplemented with 10% FBS (Euroclone, Italy), 100 U/mL penicillin, and 100 mg/mL streptomycin (Euroclone, Italy) at 37 °C in humidified air with 5% CO_2_.

### 4.3. Pharmacological Treatments on C2C12 Cells

Upon reaching confluence, myoblasts were plated in proper cell culture plates according to experimental procedures. After 24 h or 48 h, they were treated with 1 µM dexamethasone (Dexa; Sigma Aldrich, Milan, Italy) for 48 h. The fractionated extracts (hexane, HEX; chloroform, CL; methanol, MET) derived from *P. phosphorea* were used in the presence of Dexa. Concentration and time of exposure for Dexa-induced damage were previously set up [20].

### 4.4. Cell Viability Assay (MTT Test) in Cell Cultures

Cell viability was evaluated by the reduction in 3-(4,5-dimethylthiozol-2-yl)-2,5-diphenyltetrazolium bromide (MTT) (Merck, Milan, Italy) as an index of mitochondrial functionality. Myoblasts were plated into 96-well cell culture plates (3 × 10^3^ cells/well). After 48 h, cells were treated with Dexa (1, 3, 10 µM) for an additional 24 or 48 h. Myoblasts were incubated with fractionated extracts of *P. phosphorea* at different concentrations (2.5, 5, 10, 25, 50 µg/mL) in combination with Dexa (1 µM) for 48 h.

J774A.1 macrophages were seeded in 96-well plates (3 × 10^4^ cells/well) containing 100 µL of medium. After 24 h, cells were treated with fractionated extracts of *P. phosphorea* at different concentrations (2, 10, 50 µg/mL) for 24 h. 5 × 10^3^ HEI-OC1 cells or 3 × 10^3^ A375 melanoma cells were seeded in 96-well plates containing 100 µL of medium. J774A.1 macrophage cells were seeded in 96-well plates (3 × 10^4^ cells/well) containing 100 µL of medium. The following day, the cells were treated in the presence or absence of different concentrations of the specific extract at different concentrations and, for HEI-OC1, a set of experiments was carried out in the presence also of 5 μM cisplatin. Treatments were carried out for 48 h. 

For all cell types, viability was measured with 3-(4,5-dimethylthiazol-2-yl)-2,5-diphenyltetrazolium bromide. Briefly, 1 mg/mL MTT in phosphate buffer saline was added to each well; then, cells were incubated for 30 min. The reaction was stopped by adding 175 µL DMSO. Then, the formazan salts were dissolved by gentle shaking for 60 min at the culture temperature specific for each cell line and quantified spectrophotometrically by reading the absorbance at 550 nm with an automatic ultra-microplate reader.

### 4.5. Immunofluorescence Staining of C2C12 Cells

C2C12 myoblasts were plated onto coverslips (5 × 10^3^ cells/slice) and grown until reaching 60% confluency. After that, cells were exposed to differentiation medium (DM, DMEM supplemented with 100 U/mL penicillin, 100 μg/mL streptomycin, 2 mM L-glutamine, and 2% horse serum) for 24 h and, subsequently, subjected to pharmacological treatment for 48 h in DM. Cells were treated with 1 µM Dexa in the presence of HEX (5, 10, 25 µg/mL) and then fixed in 4% buffered paraformaldehyde for 10 min at room temperature. Fixed cells were permeabilized with PBS containing 0.1% Triton X-100 for 10 min and then incubated with a blocking solution containing 0.1% Triton X and 0.5% BSA in PBS for 30 min. After blocking, cells were incubated at 4 °C overnight with mouse monoclonal anti-myogenin (1:40; Santa Cruz Biotechnology, CA, USA). To reveal the immunostaining, the cells were incubated with goat anti-mouse Alexa Fluor 488-conjugated IgG (1:500; Life Technologies, Italy) for 2 h at room temperature. Negative controls were carried out by replacing the primary antibody with non-immune mouse serum; cross-reactivity of the secondary antibody was tested in control experiments in which the primary antibody was omitted. During some experiments, counterstaining was performed with either TRITC-labeled phalloidin (1:40; Life Technologies, Italy) to reveal filamentous actin and 40,6-diamidine-20-phenylindole dihydrochloride (DAPI) (1:2000; Merck, Milan, Italy) to reveal nuclei. After washing, the coverslips containing the immunolabeled cells were mounted with the mounting medium ProLong (Life Technologies, Milan, Italy) and observed under a motorized Leica DM6000B microscope equipped with a DFC350FX camera (Leica, Mannheim, Germany). Quantitative analysis of myogenin-positive cells was performed by collecting at least four independent fields through a 40X 0.5NA objective. Myogenin-positive cells were counted in 72 h differentiated myotubes using the “cell counter” plugin of ImageJ. The myogenin signal in immunostained sections was quantified using FIJI software (distributed by ImageJ v. 1.54f, NIH, Bethesda, MD, USA) by automatic thresholding images with the aid of the “Moments” algorithm, which we found to provide the most consistent pattern recognition across all acquired images. Results were expressed as a percentage calculated by the ratio between the number of myogenin-positive cells and the total cells identified by DAPI (100%).

### 4.6. Proliferation Analysis Through CFSE Assay on C2C12 Cells

For proliferation analysis with carboxyfluorescein succinimidyl ester (CFSE, Life Technologies, Milan, Italy) cells were grown in 6-well plates (3.5 × 10^4^ cells/well) for 24 h and, subsequently, treated with HEX (5, 10, 25 µM), MET (25 µM) and CL (10 µM) in combination with 1 µM Dexa for 24 and 48 h. Briefly, after treatment, attached cells were collected, washed with PBS, harvested with 0.25% trypsin, and centrifuged at 1200 RPM for 5 min at room temperature. The supernatant was carefully discarded, and pellets were resuspended in 900 µL of PBS. The variation in the proliferation index, at 24 and 48 h, was performed to study cell proliferation using a flow cytometer (CyFLow Space flowcytometer (Sysmex Partec, Goerlitz, Germany).

### 4.7. Apoptosis Analysis of C2C12 Cells by Flow Cytometry

To quantitatively evaluate the degree of apoptosis, annexin V-fluorescein isothiocyanate and propidium iodide (PI) double staining was performed using the Annexin V Apoptosis Detection Kit (Santa Cruz Biotechnology, CA, USA). To this end, C2C12 myoblasts were grown in 6-well plates (7 × 10^4^ cells/well) for 24 h and, subsequently, treated with HEX (5, 10, 25 µM) in combination with starvation (serum-free DMEM) for 48 h. Briefly, after treatment, floating cells were collected, whereas adherent ones were washed with PBS and harvested with 0.25% trypsin. Cells were then centrifuged at 1200 RPM for 5 min at room temperature. The supernatant was carefully discarded, and pellets were resuspended in 1 mL PBS and centrifuged as before. The pelleted cells were then resuspended in 100 µL of the binding buffer (double-distilled water+ HEPES 10 mM, NaCl 140 mM, and CaCl_2_ 2 mM) containing 1 µg Annexin V-FITC and 0.5 µg propidium iodide and incubated for 10–15 min at room temperature. After the incubation period, 800 µL of binding buffer was added to each tube. Fluorescence was measured immediately using a flow cytometer (CyFLow Space flowcytometer (Sysmex Partec, Goerlitz, Germany). Finally, the percentage of Annexin V-positive and propidium iodide (PI) cells was calculated.

### 4.8. Pharmacological Treatment and Nitrite Measurement in J774A.1 Macrophages

The effect of HEX, MET, and CL extracts of *P. phosphorea* on the nitric oxide (NO) production was assessed by measuring nitrites, stable metabolites of NO, in macrophage medium via Griess reaction. J774A.1 macrophages (2.5 × 10^5^ cells per well seeded in a 24-well plate) were incubated in non-cytotoxic concentrations (2, 10, 50 µg/mL) of fractionated extracts of *P. phosphorea* for 30 min, and subsequently with LPS (1 μg/mL) for 24 h. Then, the cell supernatant was collected and incubated with 100 µL of Griess reagent (0.2% naphthylethylenediamine dihydrochloride and 2% sulphanilamide in 5% phosphoric acid) at room temperature for 10 min in order to allow the formation of a colored azo dye. The absorbance was read at 550 nm. Serial-diluted sodium nitrite (Sigma-Aldrich, St. Louis, MO, USA) was used to generate a standard curve. The data were expressed as µM of nitrite. Each sample was determined in triplicate.

### 4.9. A375 Cell Extract Preparation and Caspase Activity Determination

A total of 3 × 10^3^ A375 cells were seeded in 100 mm diameter plates and incubated both in the presence and in the absence of the extract. After 48 h incubation, cells were scraped off, collected and centrifuged at 700× *g* for 5 min at 4 °C. Supernatants were discarded and 50 μL of lysis buffer containing 150 mM NaCl, 0.5 mM EGTA, 0.5 mM EDTA, 1% Triton X-100, and proteinase inhibitor cocktail (Calbiochem, San Diego, CA, USA) in 20 mM Tris-Cl pH 7.4, was added. Vials with cells and lysis buffer were then vortexed for 1 min and kept on ice for 30 min prior to centrifugation at 10,000× *g* for 15 min at 4 °C. The supernatants were collected, and the concentration of protein extracts was determined by the Bradford method. Aliquots were kept at −80 °C and used to measure caspase activity.

Determination of the activity of caspase-3 was carried out in a 96-well plate in a total volume of 100 µL of 50 mM Tris-HCl pH, 10 mM dithiothreitol, using 100 µg protein and 200 µM of the tetrapeptide DEVD-conjugated to para-nitroaniline (pNA) for 4 h at 37 °C (Cayman Chemical, Ann Arbor, MI, USA). The released pNA was measured in a spectrophotometer at 405 nm every 30 min (EL 808 (Bio-Tek Instruments Inc., Winooski, VT, USA). Caspase-3 activity was measured as the variation in absorbance/time and normalized to the values of control cells.

### 4.10. Statistical Analysis

Statistical analysis of the data obtained with C2C12 cells was performed using a non-parametric *t*-test. Statistical analysis of the data obtained with HEI-OC1 cells, Student’s *t*-test, or one-way ANOVA, followed by Tukey’s multiple comparisons test, was used. 

A *p*-value < 0.05 was set as statistically significant. Data were analyzed using “GraphPad Prism 10” software (GraphPad Software Inc., San Diego, CA, USA).

### 4.11. Liquid Chromatography–High-Resolution Tandem Mass Spectrometry (LC-HRMS^2^)

All *P. phosphorea* extracts were dissolved in MeOH at a concentration of 1 mg/mL for untargeted LC-HRMS^2^ analyses. High-resolution ESI-MS data were recorded on a Thermo Scientific Q Exactive Focus Orbitrap combined with Thermo Ultimate 3000 HPLC system. A Hypersil C18 column (100 × 4.6 mm, 3 μm) kept at 25 °C, an elution gradient of 0.1% HCOOH in H_2_O (eluent A) and 0.1% HCOOH in ACN (eluent B), and a flow rate of 400 µL/min were used. The gradient program was set as follows: 10% B for 1 min, 10–100% B over 30 min, and 100% B for 10 min. The volume injected was set at 5 µL. Mass spectra were acquired both in positive and negative ion detection modes. In positive ion detection mode, MS parameters were as follows: a spray voltage of 4.80 kV, a capillary temperature of 285 °C, a sheath gas rate of 32 units N_2_, an auxiliary gas rate of 15 units N_2,_ an S-lens RF level of 55 and an auxiliary gas heater temperature of 150 °C. While in negative ion detection mode, a spray voltage of 3.20 kV, a capillary temperature of 285 °C, a sheath gas rate of 45 units N_2_, an auxiliary gas rate of 10 units N_2_, an S-lens RF level of 55, and an auxiliary gas heater temperature of 150 °C were set. An MS scan range of *m*/*z* 150–2000 was selected with a resolution of 70,000 and an AGC target of 1 × 10^6^. HRMS^2^ spectra were acquired in data-dependent acquisition (DDA) mode at a resolution of 70,000 and an AGC target of 5 × 10^4^, setting three MS^2^ events after each full MS scan. HRMS^2^ scans were achieved for selected ions with high-energy collisional dissociation (HCD) fragmentation, an isolation width of 2.00 Da, a normalized collision energy of 15 and 30 units, and an automated injection time.

Mass data were analyzed using the Thermo FreeStyle™ 1.8 SP2 software version 1.8.63.0 (Thermo Fisher Scientific Inc., Waltham, MA, USA).

### 4.12. LC-HRMS^2^ Data Processing and Molecular Networking

LC-HRMS^2^ data of *P. phosphorea* extracts (hexanic, CHCl_3,_ MeOH) were preprocessed separately based on the ESI ion detection mode (positive and negative), aiming to obtain two distinct molecular networks. First, MS raw LC-HRMS^2^ data were processed in batch mode with the software MZmine version 2.53. Briefly, after uploading MS raw files into MZmine 2.53 [82], mass detection was performed on centroid data, setting the noise level at 100,000 and 1000 to the mass level 1 and the mass level 2, respectively. FTMS shoulder peaks were removed by applying the Lorentzian extended peak model at a resolution of 30,000. Chromatograms were built using the ADAP chromatogram builder setting at least 5 consecutive scans in the chromatogram, a minimum height of 100,000, a group intensity threshold of 100,000, and an *m*/*z* tolerance of 0.01 Da or 10 ppm. The smoothing algorithm was performed, defining a filter width of 7. The chromatograms were deconvoluted using the local minimum feature resolver, specifying a chromatographic threshold of 10%, the search of minimum in a range of 0.2 min, the minimum relative height and the minimum absolute height at 5% and 100,000, respectively, the minimum ratio between peak top intensity and side data points at 1.3, and maximum peak duration at 10 min. In addition, an *m*/*z* range of 0.01 Da and an RT range of 0.5 min for MS^2^ scan pairing were chosen. Finally, peak lists were filtered, setting a chromatographic FWHM range of 1.50. The alignment of peaks was conducted with the join aligner method, fixing an *m*/*z* tolerance of 0.01 (or 5 ppm), an absolute RT tolerance of 0.2 min, and a score weight of *m*/*z* and retention time at 50 and 50, respectively. [M+Na–H], [M+K–H], [M+Mg−2H], [M+NH_3_], [M-Na+NH_4_], [M+1, ^13^C] adducts were filtered out by setting the maximum relative height at 100%. Peaks without associated MS^2^ spectra were filtered out from the peak list. The feature list spectra were then exported into an .mgf file, while the table of quantification was exported as a .csv file and submitted to the FBMN workflow on the Global Natural Product Social Molecular Networking (GNPS) platform [40,83]. A network was then generated with the following parameters: precursor ion *m*/*z* tolerance 0.02 Da, fragment ion *m*/*z* tolerance 0.05 Da, cosine score ≥ 0.7, minimum matched peaks = 4, maximum number of neighbor nodes = 10, maximum number of nodes in a single network = 100. The spectra in the network were searched against GNPS spectral libraries. A cosine score above 0.7 and at least 6 matching peaks were required to keep matches between network spectra and library spectra. Generated networks were visualized using Cytoscape version 3.9.1 [84].

Chromatographic data in the .csv file were mapped to the relevant nodes in the generated network (available at https://gnps.ucsd.edu/ProteoSAFe/status.jsp?task=a6456b0e09da4188b9226f5d7f6800ad and https://gnps.ucsd.edu/ProteoSAFe/status.jsp?task=296e224cfe8a457d8e76185e0f1da280 accessed on 26 August 2024). MS tandem spectra of all compounds reported in the molecular network can be found by accessing the GNPS link. Additional compound annotations were performed by searching putative molecular formulae against databases such as LIPIDMAPS (https://www.lipidmaps.org/tools/ms/LMSD_search_mass_options.php, accessed on 26 August 2024), Pubchem (https://pubchem.ncbi.nlm.nih.gov/, accessed on 26 August 2024), Metabolomics workbench (https://www.metabolomicsworkbench.org/search/ms.php, accessed on 26 August 2024).

## Figures and Tables

**Table 1:** Glycerophosphoinositols (GPIs) identified in the organic extracts from *P. phosphorea*.

	Compound ^a^	Rt (min.)	[M+H]^+^ (*m*/*z*)	[M−H]^−^ (*m*/*z*)
Monoacyl GPIs	LPI 16:0 ^b^	20.8	-	C_25_H_48_O_12_P (571.2888)
	LPI 17:0	21.9	-	C_26_H_50_O_12_P (585.3048)
	LPI 18:0 ^b^	23.0	C_27_H_54_O_12_P (601.3344)	C_27_H_52_O_12_P (599.3202)
	LPI 18:1 ^b^	21.2	-	C_27_H_50_O_12_P (597.3049)
	LPI 19:0	24.2	-	C_28_H_54_O_12_P (613.3360)
	LPI 20:0 ^b^	25.4	-	C_29_H_56_O_12_P (627.3517)
	LPI 20:1	23.3	-	C_29_H_54_O_12_P (625.3359)
Monoalkyl GPIs	LPI O-16:0	21.2	C_25_H_52_O_11_P (559.3239)	C_25_H_50_O_11_P (557.3096)
	LPI O-17:0	22.4	C_26_H_54_O_11_P (573.3395)	C_26_H_52_O_11_P (571.3252)
	LPI O-18:0	23.5	C_27_H_56_O_11_P (587.3554)	C_27_H_54_O_11_P (585.3412)
	LPI O-18:1	22.2	-	C_27_H_52_O_11_P (583.3256)
Oxidized GPIs	PI 18:0/5:1;O2	23.6	-	C_33_H_62_O_14_P (713.352)
	PI 18:0/6:1;O	24.6	-	C_33_H_60_O_14_P (711.3727)
	PI 18:0/6:1;O2	23.8	-	C_33_H_60_O_15_P (727.3677)
	PI 18:0/7:1;O	25.1	-	C_34_H_62_O_14_P (725.3888)
	PI 19:0/6:1;O2	24.1/24.9	-	C_34_H_62_O_15_P (741.3835)
	PI 20:0/6:1;O2	26.1	-	C_35_H_64_O_15_P (755.3991)
	PI 20:1/6:1;O2	23.9	-	C_35_H_62_O_16_P (753.3829)
	PI 18:0/9:2;O3	23.2	-	C_36_H_64_O_16_P (783.3939)
	PI O-18:0/5:1;O2	24.2	-	C_32_H_60_O_14_P (699.3730)
	PI O-18:0/6:1;O2	24.5	-	C_33_H_62_O_14_P (713.3882)

^a^ Compounds are referred to by the LIPID MAPS abbreviations [26]; ^b^ Compounds reported in the LIPID MAPS database.

**Table 2:** Glycerophosphoglycerols (GPGs) identified in the organic extracts from *P. phosphorea*.

	Compound ^a^	Rt (min.)	[M+H]^+^ (*m*/*z*)	[M−H]^−^ (*m*/*z*)
Monoacyl GPGs	LPG 16:0 ^b^	22.3	-	C_22_H_44_O_9_P (483.2732)
	LPG 18:0 ^b^	24.9	-	C_24_H_48_O_9_P (511.3046)
	LPG 18:1 ^b^	22.6	-	C_24_H_46_O_9_P (509.2887)
Monoalkyl GPGs	LPG O-14:0	20.5	C_20_H_44_O_8_P (443.2779)	C_20_H_42_O_8_P (441.2623)
	LPG O-15:0	21.8	C_21_H_46_O_8_P (457.2930)	C_21_H_44_O_8_P (455.2783)
	LPG O-16:0	23.1/23.5	C_22_H_48_O_8_P (471.3095)	C_22_H_46_O_8_P (469.2936)
	LPG O-16:1	21.0	-	C_22_H_44_O_8_P (467.2781)
	LPG O-17:0	24.5	C_23_H_50_O_8_P (485.3252)	C_23_H_48_O_8_P (483.3097)
	LPG O-18:0	26.1	C_24_H_52_O_8_P (499.3394)	C_24_H_50_O_8_P (497.3255)
Oxidized GPGs	LPG 18:1;O	17.9	-	C_24_H_46_O_10_P (525.2834)

^a^ Compounds are referred to by the LIPID MAPS abbreviations [26]; ^b^ Compounds reported in the LIPID MAPS database.

**Table 3:** Glycerophosphoethanolamines (GPEs) identified in the organic extract from *P. phosphorea*.

	Compound ^a^	Rt (min.)	[M+H]^+^ (*m*/*z*)	[M−H]^−^ (*m*/*z*)
Monoacyl GPEs	LPE 16:1 ^b^	27.6	C_21_H_43_NO_7_P (452.2768)	C_21_H_41_NO_7_P (450.2627)
	LPE 18:1 ^b^	25.0	-	C_23_H_45_NO_7_P (478.2939)

^a^ Compounds are referred to by the LIPID MAPS abbreviations [26]; ^b^ Compounds reported in the LIPID MAPS database.

**Table 4:** Glycerophosphates (GPs) identified in the organic extract from *P. phosphorea*.

	Compound ^a^	Rt (min.)	[M+H]^+^ (*m*/*z*)	[M−H]^−^ (*m*/*z*)
Monoacyl GPs	LPA 16:1 ^b^	26.0	-	C_16_H_36_O_7_P (407.2204)
	LPA 18:0 ^b^	31.1/31.8	-	C_21_H_42_O_7_P (437.2674)
	LPA 18:1 ^b^	30.5	-	C_21_H_40_O_7_P (435.2515)
	LPA 20:1	31.7	-	C_23_H_44_O_7_P (463.2830)
Monoalkyl GPs	LPA O-16:0	29.2	C_19_H_42_O_6_P (397.2717)	-
Cyclic monoacyl GPs	CPA 16:0 ^b^	24.7	-	C_19_H_36_O_6_P (391.2256)
	CPA 16:1	23.9	-	C_19_H_34_O_6_P (389.2098)
	CPA 18:0 ^b^	30.0	-	C_21_H_40_O_6_P (419.2569)

^a^ Compounds are referred to by the LIPID MAPS abbreviations [26]; ^b^ Compounds reported in the LIPID MAPS database.

**Table 5:** Glycerophosphoserine (GPS) identified in the organic extract from *P. phosphorea*.

	Compound ^a^	Rt (min.)	[M+H]^+^ (*m*/*z*)	[M−H]^−^ (*m*/*z*)
Monoacyl GPS	LPS 18:0 ^b^	29.6	C_24_H_49_NO_9_P (526.3139)	C_24_H_47_NO_9_P (524.2994)

^a^ Compounds are referred to by the LIPID MAPS abbreviations [26]; ^b^ Compounds reported in the LIPID MAPS database.

**Table 6:** Glycerophosphocholines (GPCs) identified in the organic extract from *P. phosphorea*.

	Compound ^a^	Rt (min.)	[M+H]^+^ (*m/z)*	[M−H]^−^ (*m*/*z*)
Monoacyl GPCs	LPC 16:0 ^b^	19.5/19.8	C_24_H_51_NO_7_P (496.3401)	-
	LPC 18:0 ^b^	22.6/23.5	C_26_H_55_NO_7_P (524.3713)	-
Oxidized GPCs	LPC 11:1;O	14.0	C_19_H_39_NO_8_P (440.2411)	-
	LPC 18:1;O	21.8/22.4	C_26_H_53_NO_8_P (538.3503)	-

^a^ Compounds are referred to by the LIPID MAPS abbreviations [26]. ^b^ Compounds reported in the LIPID MAPS database.

**Table 7:** Ceramide phosphoinositols (Ceramide PIs) identified in the organic extract from *P. phosphorea*.

	Compound ^a^	Rt (min.)	[M+H]^+^ (*m*/*z*)	[M−H]^−^ (*m*/*z*)
Ceramide PIs	IPC 24:2;O3	31.1	-	C_30_H_55_NO_12_P (652.3469)
	IPC 33:1;O2	30.9	-	C_39_H_75_NO_11_P (764.5082)
	IPC (d18:1/16:0)	32.8	C_40_H_79_NO_11_P (780.5378)	C_40_H_77_NO_11_P (778.5240)
	IPC (d18:2/16:0)	31.0	C_40_H_77_NO_11_P (778.5229)	C_40_H_75_NO_11_P (776.5082)
	IPC 34:3;O2	29.7	-	C_40_H_73_NO_11_P (774.4928)
	IPC 35:2;O2	32.3	-	C_41_H_77_NO_11_P (790.5240)

^a^ Compounds are referred to by the LIPID MAPS abbreviations [26].

**Table 8:** Prostaglandins identified in the organic extracts from *P. phosphorea*.

	Compound ^a^	Rt (min.)	[M+H]^+^ (*m*/*z*)	[M−H]^−^ (*m*/*z*)
Prostaglandins	FA 20:5;O2	23.8	-	C_20_H_29_O_4_ (333.2073)
	FA 20:4;O2	25.8	-	C_20_H_31_O_4_ (335.2232)
	FA 20:4;O3	19.0/19.7/20.0	-	C_20_H_31_O_5_ (351.2178)

^a^ Compounds are referred to by the LIPID MAPS abbreviations [26].

## Data Availability

The molecular networks and mass spectrometry data are available athttps://gnps.ucsd.edu/ProteoSAFe/status.jsp?task=a6456b0e09da4188b9226f5d7f6800ad and https://gnps.ucsd.edu/ProteoSAFe/status.jsp?task=296e224cfe8a457d8e76185e0f1da280 accessed on 26 August 2024.

## Supplementary Materials

The following supporting information can be downloaded at https://www.mdpi.com/article/10.3390/md23050218/s1, Figure S1: Full molecular network generated from LC-MS/MS analyses of the *Pennatula phosphorea* extract acquired in the positive ion detection mode.; Figure S2. Full molecular network generated from LC-MS/MS analyses of the *Pennatula phosphorea* extract acquired in the negative ion detection mode.; Figure S3. HR ESI-MS^2^ spectrum of the [M−H]^−^ ion of a representative monoacyl glycerophosphoglycerol (GPG) from *Pennatula phosphorea* (LPG 18:0); Figure S4. HR ESI-MS^2^ spectrum of the [M−H]^−^ ion of a representative cyclic monoacyl glycerophosphate from *Pennatula phosphorea* (CPA 16:0); Figure S5. HR ESI-MS^2^ spectrum of the [M+H]^+^ (A) and [M−H]^−^ (B) ions of the putative monoacyl glycerophosphoserine from *Pennatula phosphorea* annotated as LPS 18:0. Figure S6. HR ESI-MS^2^ spectrum of the [M+H]^+^ ion of the putative monoacyl glycerophosphocholine from *Pennatula phosphorea* annotated as LPC 18:0. Table S1. Annotated compounds and their relative abundance in the organic extracts from *P. phosphorea*. Table S2. Compounds detected in positive ion detection mode annotated in the LIPID MAPS database. Table S3. Compounds detected in negative ion detection mode annotated in the LIPID MAPS database.

## References

[1] Fabricius K., Hopley D. (2011). Octocorallia. Encyclopedia of Modern Coral Reefs: Structure, Form and Process.

[2] Daly M., Brugler M., Cartwright P., Collins A., Dawson M., Fautin D., France S., McFadden C., Opresko D., Rodriguez E. (2006). The Phylum Cnidaria: A Review of Phylogenetic Patterns and Diversity 300 Years After Linnaeus. Zootaxa.

[3] Changyun W., Haiyan L., Changlun S., Yanan W., Liang L., Huashi G. (2008). Chemical defensive substances of soft corals and gorgonians. Acta Ecol. Sin..

[4] Raimundo I., Silva S.G., Costa R., Keller-Costa T. (2018). Bioactive Secondary Metabolites from Octocoral-Associated Microbes—New Chances for Blue Growth. Mar. Drugs.

[5] Aceret T.L., Coll J.C., Uchio Y., Sammarco P.W. (1998). Antimicrobial activity of the diterpenes flexibilide and sinulariolide derived from *Sinularia flexibilis* Quoy and Gaimard 1833 (Coelenterata: Alcyonacea, Octocorallia). Comp. Biochem. Physiol. C Pharmacol. Toxicol. Endocrinol..

[6] Lin Y.S., Khalil A.T., Chiou S.H., Kuo Y.C., Cheng Y.B., Liaw C.C., Shen Y.C. (2008). Bioactive marine prostanoids from octocoral *Clavularia viridis*. Chem. Biodivers..

[7] Duh C.Y., El-Gamal A.A., Chu C.J., Wang S.K., Dai C.F. (2002). New cytotoxic constituents from the Formosan soft corals *Clavularia viridis* and *Clavularia violacea*. J. Nat. Prod..

[8] Cairns S. (2007). Deep-water corals: An overview with special reference to diversity and distribution of deep-water Scleractinian corals. Bull. Mar. Sci..

[9] Sharifi S., Ghavam Mostafavi P., Mashinchian Moradi A., Givianrad M.H., Niknejad H. (2018). Inducing Apoptosis of Cancer Cells Using Sea Pen *Virgularia gustaviana* Extract Which is Comparable to Cembrane Diterpene Sarcophine. Iran. J. Pharm. Sci..

[10] Williams G.C. (2011). The global diversity of sea pens (Cnidaria: Octocorallia: Pennatulacea). PLoS ONE.

[11] Bastari A., Pica D., Ferretti F., Micheli F., Cerrano C. (2018). Sea pens in the Mediterranean Sea: Habitat suitability and opportunities for ecosystem recovery. IJMS.

[12] Porporato E.M.D., Mangano M.C., De Domenico F., Giacobbe S., Spanò N. (2014). First observation of *Pteroeides spinosum* (Anthozoa: Octocorallia) fields in a Sicilian coastal zone (Central Mediterranean Sea). Mar. Biodivers..

[13] Thomas S.A.L., Sanchez A., Kee Y., Wilson N.G., Baker B.J. (2019). Bathyptilones: Terpenoids from an Antarctic Sea Pen, *Anthoptilum grandiflorum* (Verrill, 1879). Mar. Drugs.

[14] Su J.-H., Huang H.-C., Chao C.-H., Yan L.-Y., Wu Y.-C., Wu C.-C., Sheu J.-H. (2005). Vigulariol, a New Metabolite from the Sea Pen *Vigularia juncea*. Bull. Chem. Soc. Jpn..

[15] Chen S.P., Sung P.J., Duh C.Y., Dai C.F., Sheu J.H. (2001). Junceol A, a new sesquiterpenoid from the sea pen *Virgularia juncea*. J. Nat. Prod..

[16] Bahl A., Jachak S., Palaniveloo K., Ramachandram T., Vairappan C., Chopra H. (2014). 2-Acetoxyverecynarmin C, a New Briarane COX Inhibitory Diterpenoid from *Pennatula aculeata*. Nat. Prod. Commun..

[17] Scarpato S., Teta R., De Cicco P., Borrelli F., Pawlik J.R., Costantino V., Mangoni A. (2023). Molecular Networking Revealed Unique UV-Absorbing Phospholipids: Favilipids from the Marine Sponge *Clathria faviformis*. Mar. Drugs.

[18] Scarpato S., Teta R., Della Sala G., Pawlik J.R., Costantino V., Mangoni A. (2020). New Tricks with an Old Sponge: Feature-Based Molecular Networking Led to Fast Identification of New Stylissamide L from *Stylissa caribica*. Mar. Drugs.

[19] Giugliano R., Della Sala G., Buonocore C., Zannella C., Tedesco P., Palma Esposito F., Ragozzino C., Chianese A., Morone M.V., Mazzella V. (2023). New Imidazolium Alkaloids with Broad Spectrum of Action from the Marine Bacterium *Shewanella aquimarina*. Pharmaceutics.

[20] Di Cesare Mannelli L., Micheli L., Lucarini E., Parisio C., Toti A., Tenci B., Zanardelli M., Branca J.J.V., Pacini A., Ghelardini C. (2020). Effects of the Combination of β-Hydroxy-β-Methyl Butyrate and R(+) Lipoic Acid in a Cellular Model of Sarcopenia. Molecules.

[21] Andrés V., Walsh K. (1996). Myogenin expression, cell cycle withdrawal, and phenotypic differentiation are temporally separable events that precede cell fusion upon myogenesis. J. Cell Biol..

[22] Chattaraj A., Syed M.P., Low C.A., Owonikoko T.K. (2023). Cisplatin-Induced Ototoxicity: A Concise Review of the Burden, Prevention, and Interception Strategies. JCO Oncol. Pract..

[23] Karimkhani C., Green A.C., Nijsten T., Weinstock M.A., Dellavalle R.P., Naghavi M., Fitzmaurice C. (2017). The global burden of melanoma: Results from the Global Burden of Disease Study 2015. Br. J. Dermatol..

[24] Domingues B., Lopes J.M., Soares P., Pópulo H. (2018). Melanoma treatment in review. Immunotargets Ther..

[25] Srivastava N., Saxena A.K. (2023). Caspase-3 Activators as Anticancer Agents. Curr. Protein. Pept. Sci..

[26] Fahy E., Subramaniam S., Murphy R.C., Nishijima M., Raetz C.R., Shimizu T., Spener F., van Meer G., Wakelam M.J., Dennis E.A. (2009). Update of the LIPID MAPS comprehensive classification system for lipids. J. Lipid Res..

[27] Della Sala G., Coppola D., Virgili R., Vitale G.A., Tanduo V., Teta R., Crocetta F., de Pascale D. (2022). Untargeted Metabolomics Yields Insights Into the Lipidome of *Botrylloides niger* Herdman, 1886, An Ascidian Invading the Mediterranean Sea. Front. Mar. Sci..

[28] Hsu F.F., Turk J., Zhang K., Beverley S.M. (2007). Characterization of inositol phosphorylceramides from *Leishmania major* by tandem mass spectrometry with electrospray ionization. J. Am. Soc. Mass Spectrom..

[29] Di Costanzo F., Di Dato V., Ianora A., Romano G. (2019). Prostaglandins in Marine Organisms: A Review. Mar. Drugs.

[30] Dasilva G., Muñoz S., Lois S., Medina I. (2019). Non-Targeted LC-MS/MS Assay for Screening Over 100 Lipid Mediators from ARA, EPA, and DHA in Biological Samples Based on Mass Spectral Fragmentations. Molecules.

[31] Lee S.H., Williams M.V., DuBois R.N., Blair I.A. (2003). Targeted lipidomics using electron capture atmospheric pressure chemical ionization mass spectrometry. Rapid Commun. Mass Spectrom..

[32] Hankin J.A., Wheelan P., Murphy R.C. (1997). Identification of Novel Metabolites of Prostaglandin E2Formed by Isolated Rat Hepatocytes. Arch. Biochem. Biophys..

[33] Yang P., Felix E., Madden T., Fischer S.M., Newman R.A. (2002). Quantitative high-performance liquid chromatography/electrospray ionization tandem mass spectrometric analysis of 2- and 3-series prostaglandins in cultured tumor cells. Anal. Biochem..

[34] Liebisch G., Fahy E., Aoki J., Dennis E.A., Durand T., Ejsing C.S., Fedorova M., Feussner I., Griffiths W.J., Köfeler H. (2020). Update on LIPID MAPS classification, nomenclature, and shorthand notation for MS-derived lipid structures. J. Lipid. Res..

[35] Greathead C., González-Irusta J.M., Clarke J., Boulcott P., Blackadder L., Weetman A., Wright P.J. (2014). Environmental requirements for three sea pen species: Relevance to distribution and conservation. IJMS.

[36] Czeczuga B. (1977). Comparative studies of carotenoids in the fauna of the Gullmar Fjord (Bohuslan, Sweden) III. Echinodermata. Hydrobiologia.

[37] Mackie A.M. (1987). Preliminary studies on the chemical defenses of the british octocorals *Alcyonium digitatum* and *Pennatula phosphorea*. Comp. Biochem. Physiol. A.

[38] Baker B.A. (2018). Efficacy of Age-Specific High-Intensity Stretch-Shortening Contractions in Reversing Dynapenia, Sarcopenia, and Loss of Skeletal Muscle Quality. J. Funct. Morphol. Kinesiol..

[39] Auclair D., Garrel D.R., Chaouki Zerouala A., Ferland L.H. (1997). Activation of the ubiquitin pathway in rat skeletal muscle by catabolic doses of glucocorticoids. Am. J. Physiol. Cell Physiol..

[40] Wang M., Carver J.J., Phelan V.V., Sanchez L.M., Garg N., Peng Y., Nguyen D.D., Watrous J., Kapono C.A., Luzzatto-Knaan T. (2016). Sharing and community curation of mass spectrometry data with Global Natural Products Social Molecular Networking. Nat. Biotechnol..

[41] Qin G.-F., Zhang X., Zhu F., Huo Z.-Q., Yao Q.-Q., Feng Q., Liu Z., Zhang G.-M., Yao J.-C., Liang H.-B. (2023). MS/MS-Based Molecular Networking: An Efficient Approach for Natural Products Dereplication. Molecules.

[42] Sikorskaya T.V. (2023). Coral Lipidome: Molecular Species of Phospholipids, Glycolipids, Betaine Lipids, and Sphingophosphonolipids. Mar. Drugs.

[43] Imbs A.B., Dembitsky V.M. (2023). Coral Lipids. Mar. Drugs.

[44] Sikorskaya T.V., Ermolenko E.V., Boroda A.V., Ginanova T.T. (2021). Physiological processes and lipidome dynamics in the soft coral *Sinularia heterospiculata* under experimental bleaching. Comp. Biochem. Physiol. B.

[45] Sikorskaya T.V., Ermolenko E.V., Imbs A.B. (2020). Effect of experimental thermal stress on lipidomes of the soft coral *Sinularia* sp. and its symbiotic dinoflagellates. J. Exp. Mar. Bio. Ecol..

[46] Sikorskaya T.V., Imbs A.B. (2018). Study of Total Lipidome of the *Sinularia siaesensis* Soft Coral. Russ. J. Org. Chem..

[47] Imbs A.B., Dang L.P.T., Rybin V.G., Svetashev V.I. (2015). Fatty Acid, Lipid Class, and Phospholipid Molecular Species Composition of the Soft Coral *Xenia* sp. (Nha Trang Bay, the South China Sea, Vietnam). Lipids.

[48] Nishikawa Y., Furukawa A., Shiga I., Muroi Y., Ishii T., Hongo Y., Takahashi S., Sugawara T., Koshino H., Ohnishi M. (2015). Cytoprotective Effects of Lysophospholipids from Sea Cucumber *Holothuria atra*. PLoS ONE.

[49] Lordan R., Redfern S., Tsoupras A., Zabetakis I. (2020). Inflammation and cardiovascular disease: Are marine phospholipids the answer?. Food Funct..

[50] Khotimchenko S.V., Vas’kovsky V.E. (2004). An Inositol-Containing Sphingolipid from the Red Alga *Gracilaria verrucosa*. Russ. J. Org. Chem..

[51] Arao K., Inagaki M., Higuchi R.J.C., Bulletin P. (1999). Constituents of Crinoidea. 1. Isolation and Structure of Inositolphosphoceramide from the Feather Star *Comanthus japonica*. Chem. Pharm. Bull..

[52] Della Sala G., Teta R., Esposito G., Pawlik J.R., Mangoni A., Costantino V. (2017). Zeamide, a Glycosylinositol Phosphorylceramide with the Novel Core Ara*p*(1β→6)Ins Motif from the Marine Sponge *Svenzea zeai*. Molecules.

[53] Bargui R., Solgadi A., Prost B., Chester M., Ferreiro A., Piquereau J., Moulin M. (2021). Phospholipids: Identification and Implication in Muscle Pathophysiology. Int. J. Mol. Sci..

[54] Wang D., Qi W., Mao X., Zhang Y., Miao Z., Zhu C., Shao Y., Ge G., Zhang W., Jin H. (2024). Gui Qi Zhuang Jin Decoction ameliorates mitochondrial dysfunction in sarcopenia mice via AMPK/PGC-1α/Nrf2 axis revealed by a metabolomics approach. Phytomedicine.

[55] Patrussi L., Mariggiò S., Corda D., Baldari C.T. (2013). The Glycerophosphoinositols: From Lipid Metabolites to Modulators of T-Cell Signaling. Front. Immunol..

[56] Chaurio R.A., Janko C., Muñoz L.E., Frey B., Herrmann M., Gaipl U.S. (2009). Phospholipids: Key Players in Apoptosis and Immune Regulation. Molecules.

[57] Ivanova M. (2020). Altered Sphingolipids Metabolism Damaged Mitochondrial Functions: Lessons Learned From Gaucher and Fabry Diseases. J. Clin. Med..

[58] Biesemann N., Ried J.S., Ding-Pfennigdorff D., Dietrich A., Rudolph C., Hahn S., Hennerici W., Asbrand C., Leeuw T., Strübing C. (2018). High throughput screening of mitochondrial bioenergetics in human differentiated myotubes identifies novel enhancers of muscle performance in aged mice. Sci. Rep..

[59] Ullah A., Bo Y., Li J., Li J., Khatun P., Lyu Q., Kou G. (2024). Insights into the Therapeutic Potential of Active Ingredients of Citri Reticulatae Pericarpium in Combatting Sarcopenia: An In Silico Approach. Int. J. Mol. Sci..

[60] Tan W.J.T., Vlajkovic S.M. (2023). Molecular Characteristics of Cisplatin-Induced Ototoxicity and Therapeutic Interventions. Int. J. Mol. Sci..

[61] Le Q., Tabuchi K., Hara A. (2016). Ceramide-1-phosphate protection of cochlear hair cells against cisplatin ototoxicity. Toxicol. Rep..

[62] Minamihata T., Takano K., Moriyama M., Nakamura Y. (2020). Lysophosphatidylinositol, an Endogenous Ligand for G Protein-Coupled Receptor 55, Has Anti-inflammatory Effects in Cultured Microglia. Inflammation.

[63] Blondeau N., Lauritzen I., Widmann C., Lazdunski M., Heurteaux C. (2002). A Potent Protective Role of Lysophospholipids Against Global Cerebral Ischemia and Glutamate Excitotoxicity in Neuronal Cultures. J. Cereb. Blood Flow Metab..

[64] Shim J.W., Jo S.H., Kim S.D., Lee H.Y., Yun J., Bae Y.-S. (2009). Lysophosphatidylglycerol inhibits formyl peptide receptor like-1-stimulated chemotactic migration and IL-1β production from human phagocytes. Exp. Mol. Med..

[65] Ohtsuki Y., Nakamura E., Asakami M., Morikawa R., Tokumura A. (2025). Inhibitory Effects of Lysophospholipids on Survival and Interleukin-8 Secretion of HT-29, a Human Colon Cancer-Derived Epithelial Cell. Biol. Pharm. Bull..

[66] Park S.-J., Im D.-S. (2021). 2-Arachidonyl-lysophosphatidylethanolamine Induces Anti-Inflammatory Effects on Macrophages and in Carrageenan-Induced Paw Edema. Int. J. Mol. Sci..

[67] Murch O., Collin M., Thiemermann C. (2007). Lysophosphatidic acid reduces the organ injury caused by endotoxemia-a role for G-protein-coupled receptors and peroxisome proliferator-activated receptor-γ. Shock.

[68] Gotoh M., Sano-Maeda K., Murofushi H., Murakami-Murofushi K. (2012). Protection of Neuroblastoma Neuro2A Cells from Hypoxia-Induced Apoptosis by Cyclic Phosphatidic Acid (cPA). PLoS ONE.

[69] Kikuchi Y., Kita T., Hirata J., Fukushima M. (1994). Preclinical studies of antitumor prostaglandins by using human ovarian cancer cells. Cancer Metastasis Rev..

[70] Jara-Gutiérrez Á., Baladrón V. (2021). The Role of Prostaglandins in Different Types of Cancer. Cells.

[71] Wang D., DuBois R.N. (2006). Prostaglandins and cancer. Gut.

[72] Panza E., Cicco P.D., Ercolano G., Armogida C., Scognamiglio G., Anniciello A.M., Botti G., Cirino G., Ianaro A. (2016). Differential expression of cyclooxygenase-2 in metastatic melanoma affects progression free survival. Oncotarget.

[73] Tadasi N., Hiroshi O. (1976). Distribution of prostaglandins in the animal kingdom. Biochim. Biophys. Acta Lipids Lipid Metab..

[74] Rowley A.F., Vogan C.L., Taylor G.W., Clare A.S. (2005). Prostaglandins in non-insectan invertebrates: Recent insights and unsolved problems. J. Exp. Biol..

[75] Di Dato V., Orefice I., Amato A., Fontanarosa C., Amoresano A., Cutignano A., Ianora A., Romano G. (2017). Animal-like prostaglandins in marine microalgae. ISME J..

[76] Weinheimer A.J., Spraggins R.L. (1969). The occurrence of two new prostaglandin derivatives (15-epi-PGA2 and its acetate, methyl ester) in the Gorgonian *Plexaura Homomalla* Chemistry of Coelenterates. XV. Tetrahedron Lett..

[77] Hurtado D.X., Castellanos F.A., Coy-Barrera E., Tello E. (2020). Prostaglandins Isolated from the Octocoral *Plexaura homomalla*: In Silico and In Vitro Studies Against Different Enzymes of Cancer. Mar. Drugs.

[78] Iguchi K., Kaneta S., Mori K., Yamada Y., Honda A., Mori Y. (1986). Bromovulone I and iodovulone I, unprecedented brominated and iodinated marine prostanoids with antitumour activity isolated from the Japanese stolonifer *Clavularia viridis* Quoy and Gaimard. J. Chem. Soc. Chem. Commun..

[79] Watanabe K., Sekine M., Takahashi H., Iguchi K. (2001). New Halogenated Marine Prostanoids with Cytotoxic Activity from the Okinawan Soft Coral *Clavularia viridis*. J. Nat. Prod..

[80] Shen Y.-C., Cheng Y.-B., Lin Y.-C., Guh J.-H., Teng C.-M., Ko C.-L. (2004). New Prostanoids with Cytotoxic Activity from Taiwanese Octocoral *Clavularia viridis*. J. Nat. Prod..

[81] Kalinec G.M., Park C., Thein P., Kalinec F. (2016). Working with Auditory HEI-OC1 Cells. J. Vis. Exp..

[82] Pluskal T., Castillo S., Villar-Briones A., Orešič M. (2010). MZmine 2: Modular framework for processing, visualizing, and analyzing mass spectrometry-based molecular profile data. BMC Bioinform..

[83] Nothias L.F., Petras D., Schmid R., Dührkop K., Rainer J., Sarvepalli A., Protsyuk I., Ernst M., Tsugawa H., Fleischauer M. (2020). Feature-based molecular networking in the GNPS analysis environment. Nat. Methods.

[84] Shannon P., Markiel A., Ozier O., Baliga N.S., Wang J.T., Ramage D., Amin N., Schwikowski B., Ideker T. (2003). Cytoscape: A software environment for integrated models of biomolecular interaction networks. Genome Res..

