# Supplementary material for: Bioactivity Assessment and Untargeted Metabolomics of the Mediterranean Sea Pen Pennatula phosphorea
Source: Mar Drugs. 2025 May 21;23(5):218. doi: 10.3390/md23050218 (PMC12113132; doi:10.3390/md23050218)
Supplement: Supplementary file 1 [file marinedrugs-23-00218-s001.zip › marinedrugs-3598194-supplementary.pdf]

marine drugs

MDPI

Supplementary information

Bioactivity Assessment and Untargeted Metabolomics of the Mediterranean Sea pen *Pennatula phosphorea*

Silvia Scarpato [1,†], Daniel Venturi [2,†], Fortunato Palma Esposito [1,*], Maria Cristina Mangano [3,4], Gianluca Sarà [4,5], Francesco Margiotta [2], Ester Pagano [6], Maria Miraglia [6], Enrico Sangiovanni [7], Mercedes Garcia-Gil [8], Lorenzo Di Cesare Mannelli [2], Carla Ghelardini [2], Mario Dell'Agli [7], Angelo A. Izzo [6], Paola Nieri [8,9], Donatella de Pascale [1] and Gerardo Della Sala [1,*]

[1] Department of Ecosustainable Marine Biotechnology, Stazione Zoologica Anton Dohrn, Via A.F. Acton, Molosiglio, 80133 Naples, Italy; silvia.scarpato@szn.it (S.S.); donatella.depascale@szn.it (D.d.P.)
[2] Department of Neuroscience, Psychology, Drug Research and Child Health-Neurofarba–Section of Pharmacology and Toxicology, University of Florence, 50139, Florence, Italy; daniel.venturi1@unifi.it (D.V.); francesco.margiotta@unifi.it (F.M.); lorenzo.mannelli@unifi.it (L.D.C.M.); carla.ghelardini@unifi.it (C.G.)
[3] Department of Integrated Marine Ecology, Stazione Zoologica Anton Dohrn, Sicily Marine Centre, Lungomare Cristoforo Colombo (Complesso Roosevelt), 90142 Palermo, Italy; mariacristina.mangano@szn.it
[4] NBFC, National Biodiversity Future Center, Piazza Marina 61, 90133 Palermo, Italy
[5] Laboratory of Ecology, Department of Earth and Marine Sciences, DiSTeM, University of Palermo, Viale delle Scienze Ed. 16, 90128 Palermo, Italy; gianluca.sara@unipa.it
[6] Department of Pharmacy, School of Medicine and Surgery, University of Naples Federico II; ester.pagano@unina.it (E.P.); maria.miraglia2@unina.it (M.M.); aaizzo@unina.it (A.A.I.)
[7] Department of Pharmacological and Biomolecular Sciences, University of Milan, Milan, Italy; enrico.sangiovanni@unimi.it (E.S.); mario.dellagli@unimi.it (M.D.)
[8] Department of Pharmacy, University of Pisa, Pisa, Italy; mercedes.garcia@unipi.it (M.G.-G.); paola.nieri@unipi.it (P.N.)
[9] Interdepartmental Center of Marine Pharmacology (MarinePHARMA), University of Pisa, Pisa, Italy
* Correspondence: fortunato.palmaesposito@szn.it (F.P.E.); gerardo.dellasala@szn.it (G.D.S.)
† These authors contributed equally to this work.

Figure S1. Full molecular network generated from LC-MS/MS analyses of the *Pennatula phosphorea* extract acquired in the positive ion detection mode.

Figure S2. Full molecular network generated from LC-MS/MS analyses of the *Pennatula phosphorea* extract acquired in the negative ion detection mode.

Figure S3. HR ESI-MS^2 spectrum of the $[M-H]^-$ ion of a representative monoacyl glycerophosphoglycerol (GPG) from *Pennatula phosphorea* (LPG 18:0).

Figure S4. HR ESI-MS^2 spectrum of the $[M-H]^-$ ion of a representative cyclic monoacyl glycerophosphate from *Pennatula phosphorea* (CPA 16:0).

Figure S5. HR ESI-MS^2 spectrum of the $[M+H]^+$ (**A**) and $[M-H]^-$ (**B**) ions of the putative monoacyl glycerophosphoserine from *Pennatula phosphorea* annotated as LPS 18:0.

Figure S6. HR ESI-MS^2 spectrum of the $[M+H]^+$ ions of a representative monoacyl glycerophosphocholine from *Pennatula phosphorea* annotated as LPC 18:0.

Table S1. Annotated compounds and their relative abundance in the organic extracts from *P. phosphorea.*

Table S2. Compounds detected in positive ion detection mode annotated in the LIPID MAPS database.

Table S3. Compounds detected in negative ion detection mode annotated in the LIPID MAPS database.

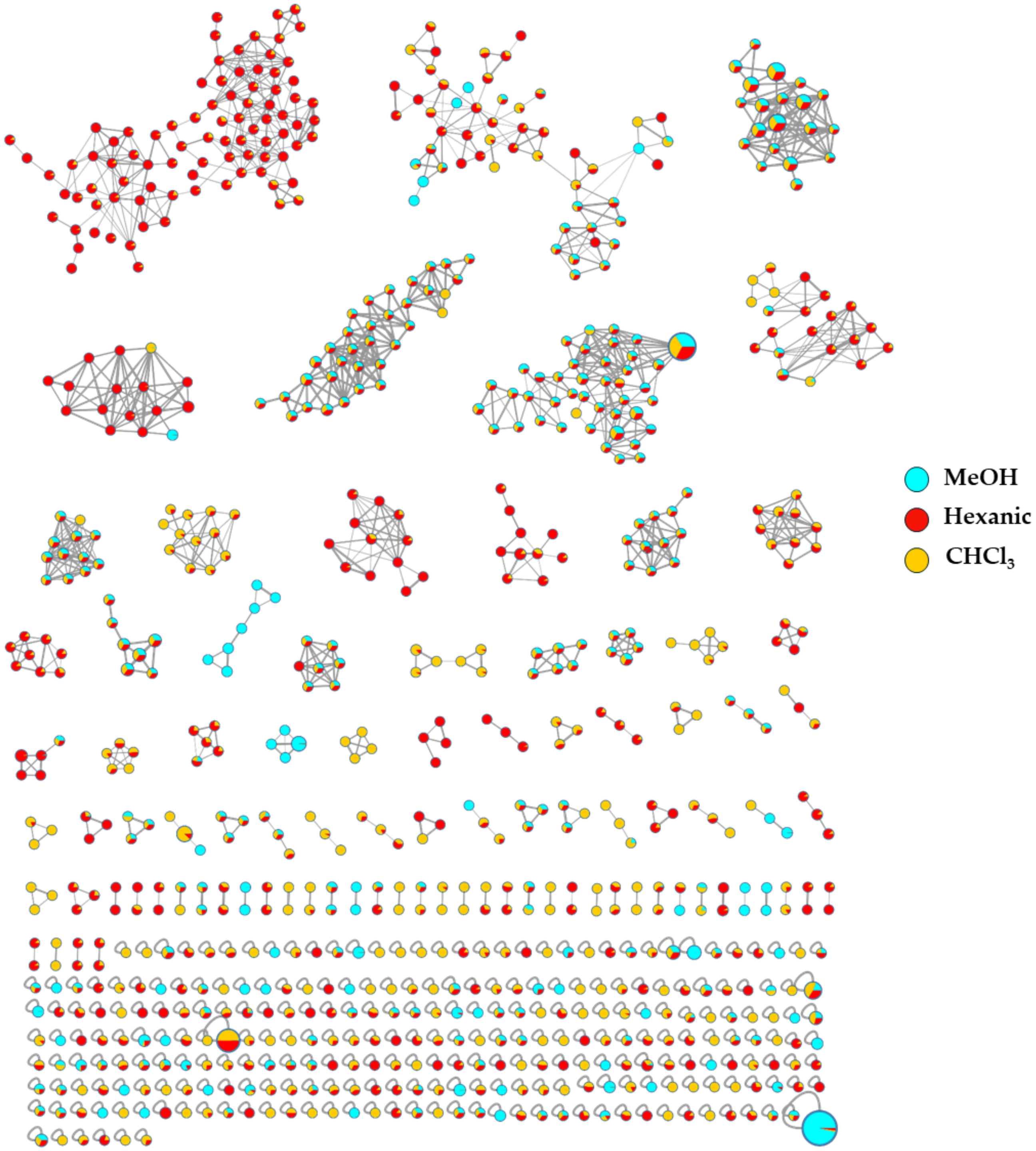

Figure S1. Full molecular network generated from LC-MS/MS analyses of the *Pennatula phosphorea* extract acquired in the positive ion detection mode. Nodes are represented as a pie chart based on the abundance of the relative precursor ion intensities in the three extracts (MeOH, hexanic, $CHCl_3$). Node size is in accordance with the peak area of the metabolite while edge thickness represents the cosine similarity of the nodes.

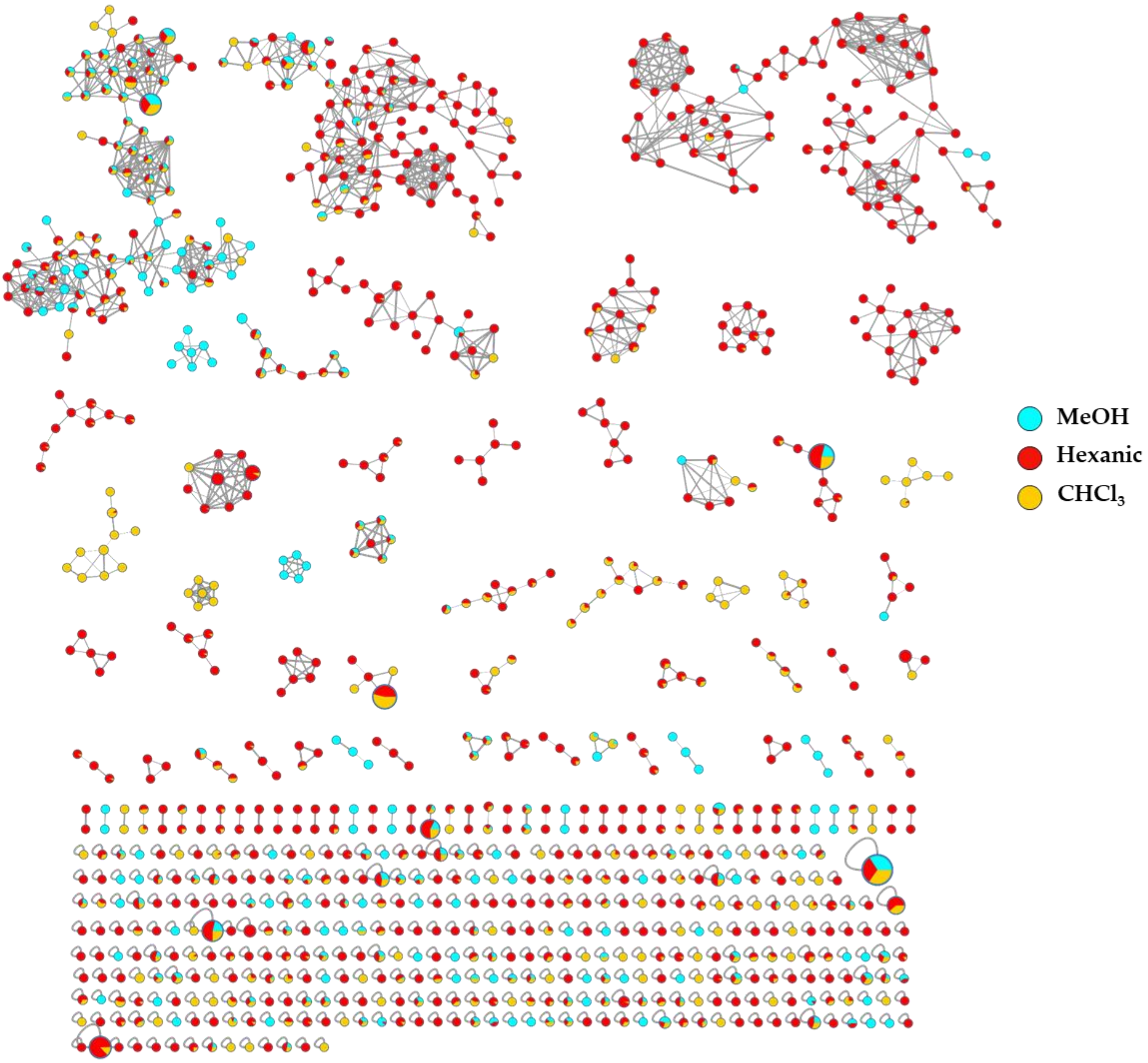

Figure S2. Full molecular network generated from LC-MS/MS analyses of the *Pennatula phosphorea* extract acquired in the negative ion detection mode. Nodes are represented as a pie chart based on the abundance of the relative precursor ion intensities in the three extracts (MeOH, hexanic, $CHCl_3$). Node size is in accordance with the peak area of the metabolite while edge thickness represents the cosine similarity of the nodes.

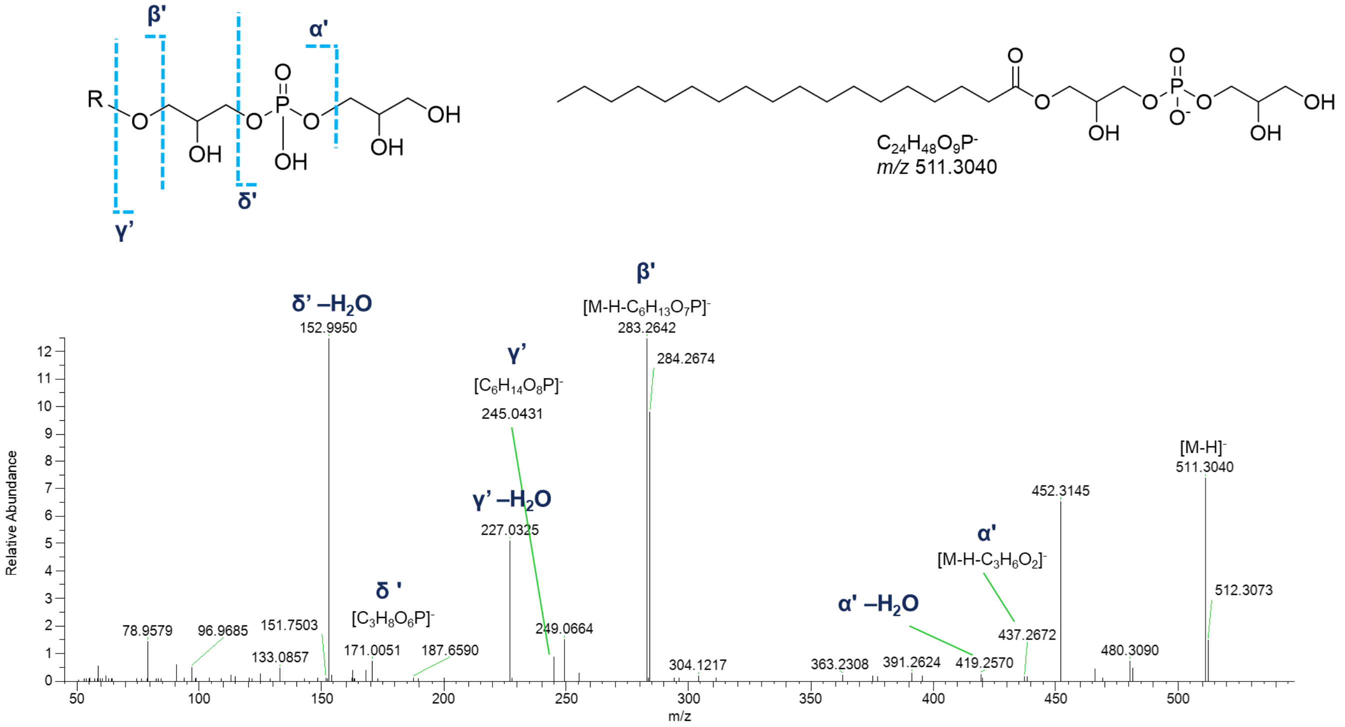

Figure S3. HR ESI-MS² spectrum of the [M-H]⁻ ion of a representative monoacyl glycerophosphoglycerol (GPG) from *Pennatula phosphorea* (LPG 18:0).

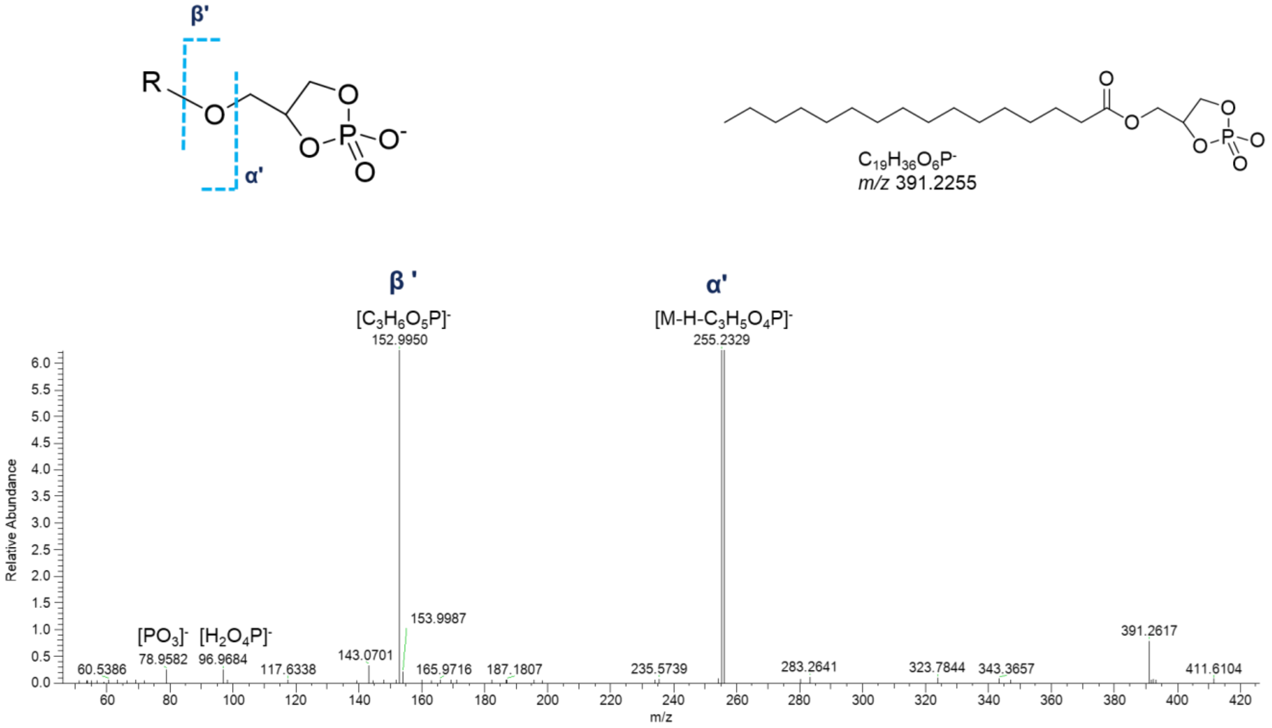

Figure S4. HR ESI-MS² spectrum of the [M-H]⁻ ion of a representative cyclic monoacyl glycerophosphate from *Pennatula phosphorea* (CPA 16:0).

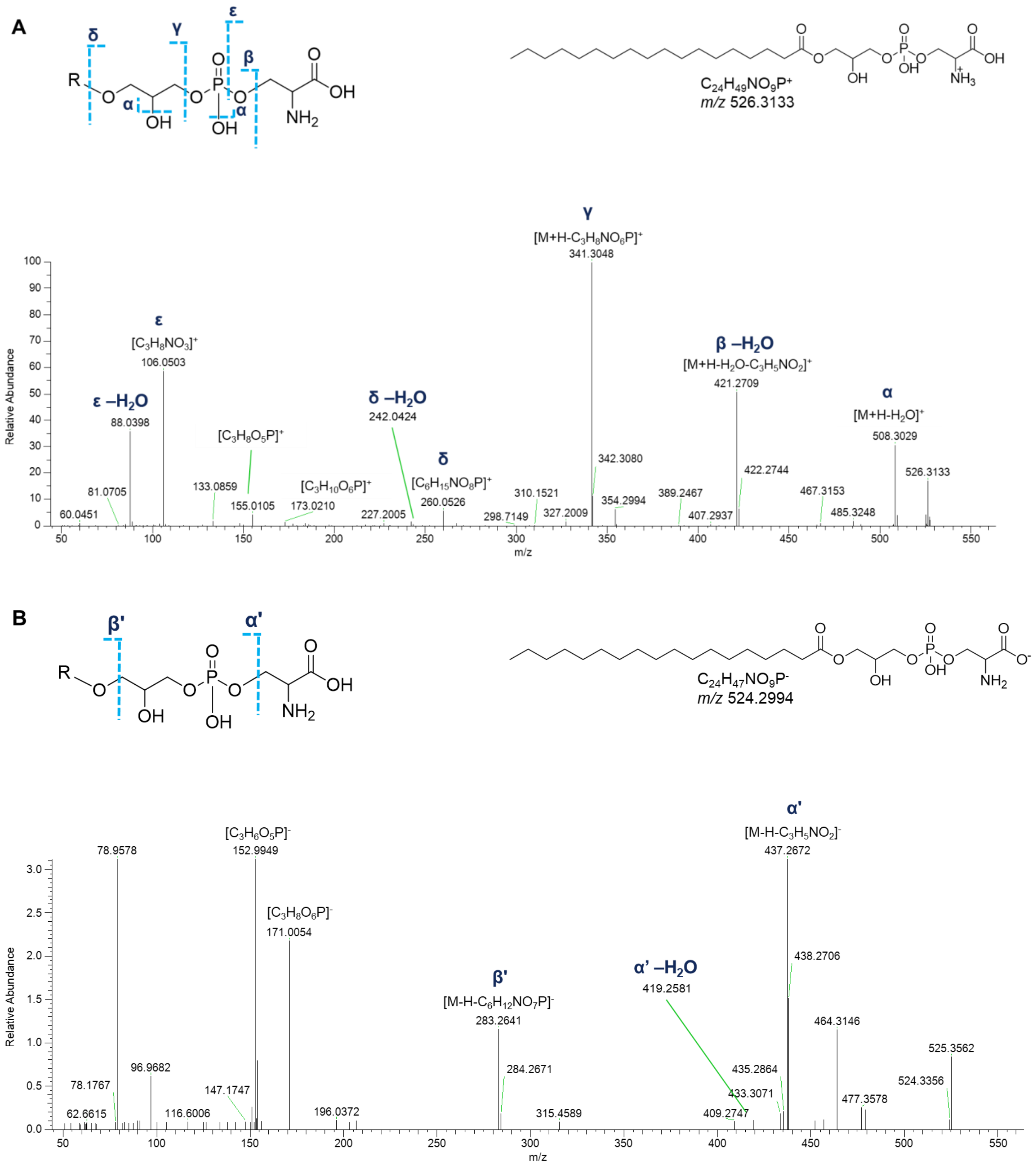

Figure S5. HR ESI-MS² spectrum of the [M+H]⁺ (**A**) and [M-H]⁻ (**B**) ions of the putative monoacyl glycerophosphoserine from *Pennatula phosphorea* annotated as LPS 18:0.

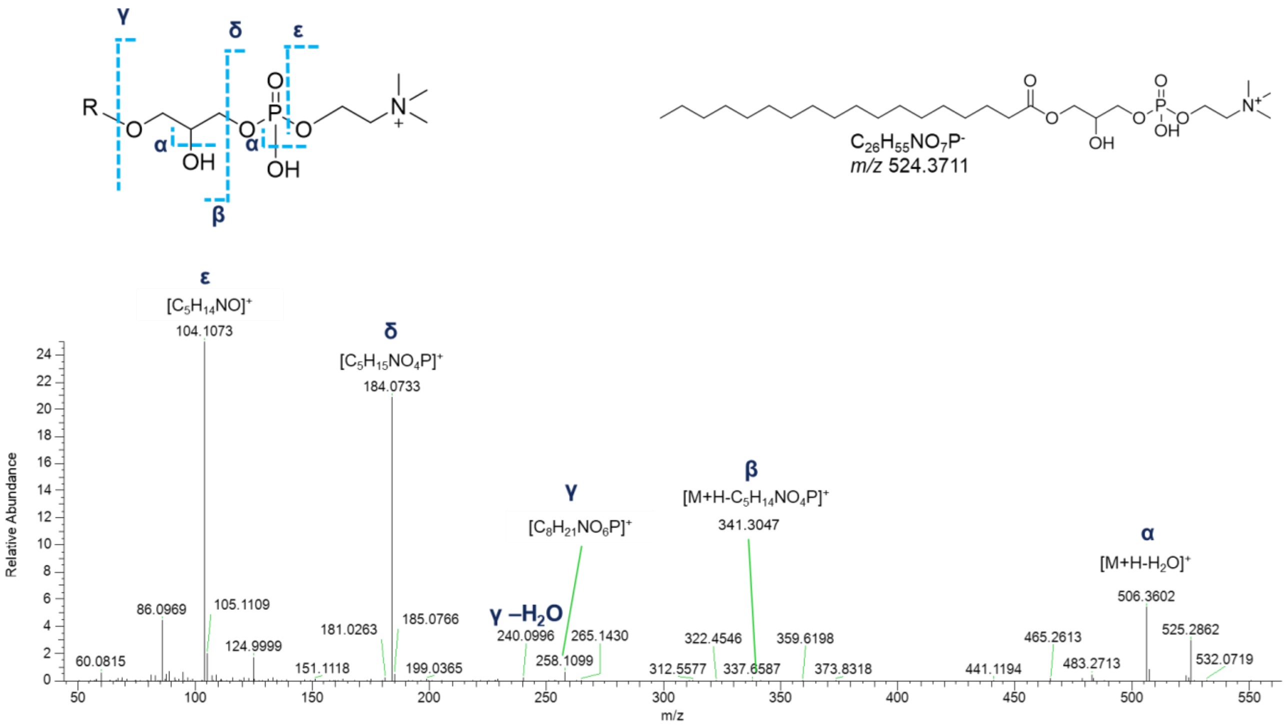

Figure S6. HR ESI-MS² spectrum of the $[M+H]^+$ ions of a representative monoacyl glycerophosphocholine from *Pennatula phosphorea* annotated as LPC 18:0.

Table S1. Annotated compounds and their relative abundance in the organic extracts from *P. phosphorea.*

	Compound [a]	Rt (min.)	[M+H]+ (m/z)	[M-H]- (m/z)	Relative abundance					
					Positive acquisition mode			Negative acquisition mode		
					MeOH	$CHCl_3$	Hexanic	MeOH	$CHCl_3$	Hexanic
**Monoacyl GPIs**	LPI 16:0	20.8	-	$C_{25}H_{48}O_{12}P$ (571.2888)	-	-	-	0.00%	0.00%	100.00%
	LPI 17:0	21.9	-	$C_{26}H_{50}O_{12}P$ (585.3048)	-	-	-	0.00%	0.00%	100.00%
	LPI 18:0	23	$C_{27}H_{54}O_{12}P$ (601.3344)	$C_{27}H_{52}O_{12}P$ (599.3202)	0%	0%	100%	0.00%	6.84%	93.16%
	LPI 18:1	21.2	-	$C_{27}H_{50}O_{12}P$ (597.3049)	-	-	-	0.00%	0.00%	100.00%
	LPI 19:0	24.2	-	$C_{28}H_{54}O_{12}P$ (613.3360)	-	-	-	0.00%	0.00%	100.00%
	LPI 20:0	25.4	-	$C_{29}H_{56}O_{12}P$ (627.3517)	-	-	-	0.00%	0.00%	100.00%
	LPI 20:1	23.3	-	$C_{29}H_{54}O_{12}P$ (625.3359)	-	-	-	0.00%	0.00%	100.00%
**Monoalkyl GPIs**	LPI O-16:0	21.2	$C_{25}H_{52}O_{11}P$ (559.3239)	$C_{25}H_{50}O_{11}P$ (557.3096)	0%	0%	100%	0.00%	0.00%	100.00%
	LPI O-17:0	22.4	$C_{26}H_{54}O_{11}P$ (573.3395)	$C_{26}H_{52}O_{11}P$ (571.3252)	0%	0%	100%	0.00%	0.00%	100.00%
	LPI O-18:0	23.5	$C_{27}H_{56}O_{11}P$ (587.3554)	$C_{27}H_{54}O_{11}P$ (585.3412)	0%	7%	93%	0.00%	5.82%	94.18%
	LPI O-18:1	22.2	-	$C_{27}H_{52}O_{11}P$ (583.3256)	-	-	-	0.00%	0.00%	100.00%
**Oxidized GPIs**	PI 18:0/5:1;O2	23.6	-	$C_{33}H_{62}O_{14}P$ (713.352)	-	-	-	0.00%	0.00%	100.00%
	PI 18:0/6:1;O	24.6	-	$C_{33}H_{60}O_{14}P$ (711.3727)	-	-	-	0.00%	0.00%	100.00%
	PI 18:0/6:1;O2	23.8	-	$C_{33}H_{60}O_{15}P$ (727.3677)	-	-	-	0.00%	0.00%	100.00%
	PI 18:0/7:1;O	25.1	-	$C_{34}H_{62}O_{14}P$ (725.3888)	-	-	-	0.00%	0.00%	100.00%
	PI 19:0/6:1;O2	24.1	-	$C_{34}H_{62}O_{15}P$ (741.3835)	-	-	-	0.00%	0.00%	100.00%
	PI 19:0/6:1;O2	24.9		$C_{34}H_{62}O_{15}P$ (741.3835)	-	-	-	0.00%	0.00%	100.00%
	PI 20:0/6:1;O2	26.1	-	$C_{35}H_{64}O_{15}P$ (755.3991)	-	-	-	0.00%	0.00%	100.00%
	PI 20:1/6:1;O2	23.9	-	$C_{35}H_{62}O_{16}P$ (753.3829)	-	-	-	0.00%	0.00%	100.00%
	PI 18:0/9:2;O3	23.2	-	$C_{36}H_{64}O_{16}P$ (783.3939)	-	-	-	0.00%	0.00%	100.00%
	PI O-18:0/5:1;O2	24.2	-	$C_{32}H_{60}O_{14}P$ (699.3730)	-	-	-	0.00%	0.00%	100.00%
	PI O-18:0/6:1;O2	24.5	-	$C_{33}H_{62}O_{14}P$ (713.3882)	-	-	-	0.00%	0.00%	100.00%
**Monoacyl GPGs**	LPG 16:0	22.3	-	$C_{22}H_{44}O_{9}P$ (483.2732)	-	-	-	0.00%	0.00%	100.00%
	LPG 18:0	24.9	-	$C_{24}H_{48}O_{9}P$ (511.3046)	-	-	-	0.00%	0.00%	100.00%

	**Compound** [a]	**Rt (min.)**	**[M+H]+ (*m*/*z*)**	**[M-H]- (*m*/*z*)**	**Relative abundance**					
					Positive acquisition mode			Negative acquisition mode		
					MeOH	$CHCl_3$	Hexanic	MeOH	$CHCl_3$	Hexanic
	LPG 18:1	22.6	-	$C_{24}H_{46}O_9P$ (509.2887)	-	-	-	0.00%	0.00%	100.00%
**Monoalkyl GPGs**	LPG O-14:0	20.5	$C_{20}H_{44}O_8P$ (443.2779)	$C_{20}H_{42}O_8P$ (441.2623)	0%	0%	100%	0.00%	0.00%	100.00%
	LPG O-15:0	21.8	$C_{21}H_{46}O_8P$ (457.2930)	$C_{21}H_{44}O_8P$ (455.2783)	0%	0%	100%	0.00%	0.00%	100.00%
	LPG O-16:0	23.1	$C_{22}H_{48}O_8P$ (471.3095)	$C_{22}H_{46}O_8P$ (469.2936)	0%	33%	67%	0.00%	10.04%	89.96%
	LPG O-16:0	23.5	$C_{22}H_{48}O_8P$ (471.3095)	$C_{22}H_{46}O_8P$ (469.2936)	0%	0%	100%	0.00%	0.00%	100.00%
	LPG O-16:1	21.0	-	$C_{22}H_{44}O_8P$ (467.2781)	-	-	-	0.00%	0.00%	100.00%
	LPG O-17:0	24.5	$C_{23}H_{50}O_8P$ (485.3252)	$C_{23}H_{48}O_8P$ (483.3097)	0%	24%	76%	0.00%	0.00%	100.00%
	LPG O-18:0	26.1	$C_{24}H_{52}O_8P$ (499.3394)	$C_{24}H_{50}O_8P$ (497.3255)	0%	12%	88%	0.00%	5.16%	94.84%
**Oxidized GPGs**	LPG 18:1;O	17.9	-	$C_{24}H_{46}O_{10}P$ (525.2834)	-	-	-	0.00%	0.00%	100.00%
**Monoacyl GPEs**	LPE 16:1	27.6	$C_{21}H_{43}NO_7P$ (452.2768)	$C_{21}H_{41}NO_7P$ (450.2627)	0%	20%	80%	0.00%	18.51%	81.49%
	LPE 18:1	25.0	-	$C_{23}H_{45}NO_7P$ (478.2939)	-	-	-	0.00%	0.00%	100.00%
**Monoacyl GPs**	LPA 16:1	26.0	-	$C_{16}H_{36}O_7P$ (407.2204)	-	-	-	0.00%	0.00%	100.00%
	LPA 18:0	31.1	-	$C_{21}H_{42}O_7P$ (437.2674)	-	-	-	0.00%	0.00%	100.00%
	LPA 18:0	31.8		$C_{21}H_{42}O_7P$ (437.2674)	-	-	-	0.00%	3.37%	96.63%
	LPA 18:1	30.5	-	$C_{21}H_{40}O_7P$ (435.2515)	-	-	-	0.00%	0.00%	100.00%
	LPA 20:1	31.7	-	$C_{23}H_{44}O_7P$ (463.2830)	-	-	-	0.00%	0.00%	100.00%
**Monoalkyl GPs**	LPA O-16:0	29.2	$C_{19}H_{42}O_6P$ (397.2717)	-	3%	10%	88%	-	-	-
**Cyclic monoacyl GPs**	CPA 16:0	24.7	-	$C_{19}H_{36}O_6P$ (391.2256)	-	-	-	0.00%	0.00%	100.00%
	CPA 16:1	23.9	-	$C_{19}H_{34}O_6P$ (389.2098)	-	-	-	0.00%	0.00%	100.00%
	CPA 18:0	28.0	-	$C_{21}H_{40}O_6P$ (419.2569)	-	-	-	0.00%	0.00%	100.00%
**Monoacyl GPS**	LPS 18:0	29.6	$C_{24}H_{49}NO_9P$ (526.3139)	$C_{24}H_{47}NO_9P$ (524.2994)	-	-	-	0.00%	10.61%	89.39%
**Monoacyl GPCs**	LPC 16:0	19.5	$C_{24}H_{51}NO_7P$ (496.3401)	-	0%	14%	86%	-	-	-
	LPC 16:0	19.8	$C_{24}H_{51}NO_7P$ (496.3401)	-	0%	0%	100%	-	-	-
	LPC 18:0	22.6	$C_{26}H_{55}NO_7P$ (524.3713)	-	0%	0%	100%	-	-	-
	LPC 18:0	23.5	$C_{26}H_{55}NO_7P$ (524.3713)	-	0%	4%	96%	-	-	-

	**Compound** [a]	**Rt (min.)**	**[M+H]⁺ (*m*/*z*)**	**[M-H]⁻ (*m*/*z*)**	**Relative abundance**					
					Positive acquisition mode			Negative acquisition mode		
					MeOH	$CHCl_3$	Hexanic	MeOH	$CHCl_3$	Hexanic
**Oxidized GPCs**	LPC 11:1;O	14.0	$C_{19}H_{39}NO_8P$ (440.2411)	-	0%	6%	94%	-	-	-
	LPC 18:1;O	21.8	$C_{26}H_{53}NO_8P$ (538.3503)	-	0%	0%	100%	-	-	-
	LPC 18:1;O	22.4	$C_{26}H_{53}NO_8P$ (538.3503)	-	0%	0%	100%	-	-	-
**Ceramide PIs**	IPC 24:2;O3	31.1	-	$C_{30}H_{55}NO_{12}P$ (652.3469)	-	-	-	0.00%	3.62%	96.38%
	IPC 33:1;O2	30.9	-	$C_{39}H_{75}NO_{11}P$ (764.5082)	-	-	-	0.00%	0.00%	100.00%
	IPC (d18:1/16:0)	32.8	$C_{40}H_{79}NO_{11}P$ (780.5378)	$C_{40}H_{77}NO_{11}P$ (778.5240)	0%	0%	100%	0.00%	0.00%	100.00%
	IPC (d18:2/16:0)	31.0	$C_{40}H_{77}NO_{11}P$ (778.5229)	$C_{40}H_{75}NO_{11}P$ (776.5082)	0%	7%	93%	0.56%	8.28%	91.21%
	IPC 34:3;O2	29.7	-	$C_{40}H_{73}NO_{11}P$ (774.4928)	-	-	-	0.00%	0.00%	100.00%
	IPC 35:2;O2	32.3	-	$C_{41}H_{77}NO_{11}P$ (790.5240)	-	-	-	0.00%	0.00%	100.00%
**Prostaglandins**	FA 20:5;O2	23.8	-	$C_{20}H_{29}O_4$ (333.2073)	-	-	-	0.00%	100.00%	0.00%
	FA 20:4;O2	25.8	-	$C_{20}H_{31}O_4$ (335.2232)	-	-	-	0.00%	100.00%	0.00%
	FA 20:4;O3	19.0	-	$C_{20}H_{31}O_5$ (351.2178)	-	-	-	0.00%	69.76%	30.24%
	FA 20:4;O3	19.7	-	$C_{20}H_{31}O_5$ (351.2178)	-	-	-	0.00%	76.60%	23.40%
	FA 20:4;O3	20.0	-	$C_{20}H_{31}O_5$ (351.2178)	-	-	-	0.00%	72.37%	27.63%

[a] Compounds are referred to by the LIPID MAPS abbreviations [26].

Table S2. Compounds detected in positive ion detection mode annotated in the LIPID MAPS database.

	Experimental Mass	Calculated Mass	Delta	Name	Formula	Ion	LIPID MAPS Status
**Monoacyl GPIs**	601.3344	601.3347	0.0003	LPI 18:0	$C_{27}H_{54}O_{12}P$	$[M+H]^+$	Curated
**Monoalkyl GPIs**	559.3239	559.3242	0.0003	LPI O-16:0	$C_{25}H_{52}O_{11}P$	$[M+H]^+$	Computationally generated
	573.3395	573.3398	0.0003	LPI O-17:0	$C_{26}H_{54}O_{11}P$	$[M+H]^+$	-
	587.3554	587.3555	0.0001	LPI O-18:0	$C_{27}H_{56}O_{11}P$	$[M+H]^+$	Computationally generated
**Monoalkyl GPGs**	443.2779	443.2768	0.0011	LPG O-14:0	$C_{20}H_{44}O_8P$	$[M+H]^+$	-
	457.2930	457.2925	0.0005	LPG O-15:0	$C_{21}H_{46}O_8P$	$[M+H]^+$	-
	471.3095	471.3081	0.0014	LPG O-16:0	$C_{22}H_{48}O_8P$	$[M+H]^+$	Computationally generated
	485.3252	485.3238	0.0014	LPG O-17:0	$C_{23}H_{50}O_8P$	$[M+H]^+$	-
	499.3394	499.3394	0	LPG O-18:0	$C_{24}H_{52}O_8P$	$[M+H]^+$	Computationally generated
**Monoacyl GPEs**	452.2768	452.2772	0.0004	LPE 16:1	$C_{21}H_{43}NO_7P$	$[M+H]^+$	Curated
**Monoalkyl GPs**	397.2717	397.2713	0.0004	LPA O-16:0	$C_{19}H_{42}O_6P$	$[M+H]^+$	Computationally generated
**Monoacyl GPS**	526.3139	526.3139	0	LPS 18:0	$C_{24}H_{49}NO_9P$	$[M+H]^+$	Curated
**Monoacyl GPCs**	496.3401	496.3398	0.0003	LPC 16:0	$C_{24}H_{51}NO_7P$	$[M+H]^+$	Curated
	524.3713	524.3711	0.0002	LPC 18:0	$C_{26}H_{55}NO_7P$	$[M+H]^+$	Curated
**Oxidized GPCs**	440.2411	440.2408	0.0003	LPC 11:1;O	$C_{19}H_{39}NO_8P$	$[M+H]^+$	-
	538.3503	538.3503	0	LPC 18:1;O	$C_{26}H_{53}NO_8P$	$[M+H]^+$	-
**Ceramide PIs**	780.5378	780.5385	0.0007	IPC (d18:1/16:0)	$C_{40}H_{79}NO_{11}P$	$[M+H]^+$	-
	778.5229	778.52290	0	IPC (d18:2/16:0)	$C_{40}H_{77}NO_{11}P$	$[M+H]^+$	-

Table S3. Compounds detected in negative ion detection mode annotated in the LIPID MAPS database.

	Experimental Mass	Calculated Mass	Delta	Name	Formula	Ion	LIPID MAPS Status
**Monoacyl GPIs**	571.2888	571.2889	0.0001	LPI 16:0	$C_{25}H_{48}O_{12}P$	$[M-H]^-$	Curated
	585.3048	585.3045	0.0003	LPI 17:0	$C_{26}H_{50}O_{12}P$	$[M-H]^-$	Computationally generated
	599.3202	599.3202	0	LPI 18:0	$C_{27}H_{52}O_{12}P$	$[M-H]^-$	Curated
	597.3049	597.3045	0.0004	LPI 18:1	$C_{27}H_{50}O_{12}P$	$[M-H]^-$	Curated
	613.336	613.3358	0.0002	LPI 19:0	$C_{28}H_{54}O_{12}P$	$[M-H]^-$	Computationally generated
	627.3517	627.3515	0.0002	LPI 20:0	$C_{29}H_{56}O_{12}P$	$[M-H]^-$	Curated
	625.3359	625.3358	0.0001	LPI 20:1	$C_{29}H_{54}O_{12}P$	$[M-H]^-$	Computationally generated
**Monoalkyl GPIs**	557.3096	557.3096	0	LPI O-16:0	$C_{25}H_{50}O_{11}P$	$[M-H]^-$	Computationally generated
	571.3252	571.3253	0.0001	LPI O-17:0	$C_{26}H_{52}O_{11}P$	$[M-H]^-$	-
	585.3412	585.3409	0.0003	LPI O-18:0	$C_{27}H_{54}O_{11}P$	$[M-H]^-$	Computationally generated
	583.3256	583.3253	0.0003	LPI O-18:1	$C_{27}H_{52}O_{11}P$	$[M-H]^-$	Computationally generated
**Oxidized GPIs**	713.352	713.3518	0.0002	PI 18:0/5:1;O2	$C_{33}H_{62}O_{14}P$	$[M-H]^-$	-
	711.3727	711.3726	0.0001	PI 18:0/6:1;O	$C_{33}H_{60}O_{14}P$	$[M-H]^-$	-
	727.3677	727.3675	0.0002	PI 18:0/6:1;O2	$C_{33}H_{60}O_{15}P$	$[M-H]^-$	-
	725.3888	725.3883	0.0005	PI 18:0/7:1;O	$C_{34}H_{62}O_{14}P$	$[M-H]^-$	-
	741.3835	741.3832	0.0003	PI 19:0/6:1;O2	$C_{34}H_{62}O_{15}P$	$[M-H]^-$	-
	755.3991	755.3988	0.0003	PI 20:0/6:1;O2	$C_{35}H_{64}O_{15}P$	$[M-H]^-$	-
	753.3829	753.3832	0.0003	PI 20:1/6:1;O2	$C_{35}H_{62}O_{16}P$	$[M-H]^-$	-
	783.3939	783.3937	0.0002	PI 18:0/9:2;O3	$C_{36}H_{64}O_{16}P$	$[M-H]^-$	-
	699.373	699.3726	0.0004	PI O-18:0/5:1;O2	$C_{32}H_{60}O_{14}P$	$[M-H]^-$	-
	713.3882	713.3883	0.0001	PI O-18:0/6:1;O2	$C_{33}H_{62}O_{14}P$	$[M-H]^-$	-
**Monoacyl GPGs**	483.2732	483.2729	0.0003	LPG 16:0	$C_{22}H_{44}O_{9}P$	$[M-H]^-$	Curated
	511.3046	511.3042	0.0004	LPG 18:0	$C_{24}H_{48}O_{9}P$	$[M-H]^-$	Curated
	509.2887	509.2885	0.0002	LPG 18:1	$C_{24}H_{46}O_{9}P$	$[M-H]^-$	Curated
**Monoalkyl GPGs**	441.2623	441.2623	0	LPG O-14:0	$C_{20}H_{42}O_{8}P$	$[M-H]^-$	-

	Experimental Mass	Calculated Mass	Delta	Name	Formula	Ion	LIPID MAPS Status
	455.2783	455.2779	0.0004	LPG O-15:0	$C_{21}H_{44}O_8P$	$[M-H]^-$	-
	469.2936	469.2936	0	LPG O-16:0	$C_{22}H_{46}O_8P$	$[M-H]^-$	Computationally generated
	467.2781	467.2779	0.0002	LPG O-16:1	$C_{22}H_{44}O_8P$	$[M-H]^-$	Computationally generated
	483.3097	483.3092	0.0005	LPG O-17:0	$C_{23}H_{48}O_8P$	$[M-H]^-$	-
	497.3255	497.3249	0.0006	LPG O-18:0	$C_{24}H_{50}O_8P$	$[M-H]^-$	Computationally generated
**Oxidized GPGs**	525.2834	525.2834	0	LPG 18:1;O	$C_{24}H_{46}O_{10}P$	$[M-H]^-$	-
**Monoacyl GPEs**	450.2627	450.2626	0.0001	LPE 16:1	$C_{21}H_{41}NO_7P$	$[M-H]^-$	Curated
	478.2939	478.2939	0	LPE 18:1	$C_{23}H_{45}NO_7P$	$[M-H]^-$	Curated
**Monoacyl GPs**	407.2204	407.2204	0	LPA 16:1	$C_{16}H_{36}O_7P$	$[M-H]^-$	Curated
	437.2674	437.2674	0	LPA 18:0	$C_{21}H_{42}O_7P$	$[M-H]^-$	Curated
	435.2515	435.2517	0.0002	LPA 18:1	$C_{21}H_{40}O_7P$	$[M-H]^-$	Curated
	463.283	463.283	0	LPA 20:1	$C_{23}H_{44}O_7P$	$[M-H]^-$	Computationally generated
**Cyclic monoacyl GPs**	391.2256	391.2255	0.0001	CPA 16:0	$C_{19}H_{36}O_6P$	$[M-H]^-$	Curated
	389.2098	389.2098	0	CPA 16:1	$C_{19}H_{34}O_6P$	$[M-H]^-$	-
	419.2569	419.2568	0.0001	CPA 18:0	$C_{21}H_{40}O_6P$	$[M-H]^-$	Curated
**Monoacyl GPS**	524.2994	524.2994	0	LPS 18:0	$C_{24}H_{47}NO_9P$	$[M-H]^-$	Curated
**Ceramide PIs**	652.3469	652.3467	0.0002	IPC 24:2;O3	$C_{30}H_{55}NO_{12}P$	$[M-H]^-$	-
	764.5082	764.5083	0.0001	IPC 33:1;O2	$C_{39}H_{75}NO_{11}P$	$[M-H]^-$	-
	778.524	778.524	0	IPC (d18:1/16:0)	$C_{40}H_{77}NO_{11}P$	$[M-H]^-$	-
	776.5082	776.5083	0.0001	IPC (d18:2/16:0)	$C_{40}H_{75}NO_{11}P$	$[M-H]^-$	-
	774.4928	774.4926	0.0002	IPC 34:3;O2	$C_{40}H_{73}NO_{11}P$	$[M-H]^-$	-
	790.524	790.5239	0.0001	IPC 35:2;O2	$C_{41}H_{77}NO_{11}P$	$[M-H]^-$	-
**Prostaglandins**	333.2073	333.2071	0.0002	FA 20:5;O2	$C_{20}H_{29}O_4$	$[M-H]^-$	-
	335.2232	335.2228	0.0004	FA 20:4;O2	$C_{20}H_{31}O_4$	$[M-H]^-$	-
	351.2178	351.2177	0.0001	FA 20:4;O3	$C_{20}H_{31}O_5$	$[M-H]^-$	-

References

26. Fahy, E.; Subramaniam, S.; Murphy, R. C.; Nishijima, M.; Raetz, C. R.; Shimizu, T.; Spener, F.; van Meer, G.; Wakelam, M. J.; Dennis , E. A. Update of the LIPID MAPS comprehensive classification system for lipids. *J. Lipid Res.* **2009,** *50 Suppl*, (Suppl), S9-14. 10.1194/jlr.R800095-JLR200
